# In-Depth Study into Polymeric Materials in Low-Density Gastroretentive Formulations

**DOI:** 10.3390/pharmaceutics12070636

**Published:** 2020-07-07

**Authors:** Nieves Iglesias, Elsa Galbis, Lucía Romero-Azogil, Elena Benito, Ricardo Lucas, M. Gracia García-Martín, M.-Violante de-Paz

**Affiliations:** Departamento de Química Orgánica y Farmacéutica, Facultad de Farmacia, Universidad de Sevilla, C/ Prof. García González, n.º 2, 41012 Seville, Spain; nievesiglesias@us.es (N.I.); elsa@us.es (E.G.); lrazogil@us.es (L.R.-A.); ebenito@us.es (E.B.); rlucas1@us.es (R.L.); graciagm@us.es (M.G.G.-M.)

**Keywords:** floating, low-density, GRDDS, raft systems, alginate, polysaccharides, cellulose, gums, Carbopol, Eudragit, gastroretentive

## Abstract

The extensive use of oral dosage forms for the treatment of diseases may be linked to deficient pharmacokinetic properties. In some cases the drug is barely soluble; in others, the rapid transit of the formulation through the gastrointestinal tract (GIT) makes it difficult to achieve therapeutic levels in the organism; moreover, some drugs must act locally due to a gastric pathology, but the time they remain in the stomach is short. The use of formulations capable of improving all these parameters, as well as increasing the resident time in the stomach, has been the target of numerous research works, with low-density systems being the most promising and widely explored, however, there is further scope to improve these systems. There are a vast variety of polymeric materials used in low-density gastroretentive systems and a number of methods to improve the bioavailability of the drugs. This works aims to expedite the development of breakthrough approaches by providing an in-depth understanding of the polymeric materials currently used, both natural and synthetic, their properties, advantages, and drawbacks.

## 1. Introduction

Although life expectancy has improved substantially in the world in recent decades, the population is aging, and its quality of life is far from optimal due to the prevalence of chronic diseases. In particular, the main difficulties encountered in achieving effective treatments are related to the transport and the ability to maintain drug concentrations within their therapeutic windows in damaged tissues.

The oral route is the most convenient and widely used method of drug administration, representing about 90% of all therapies used [1]. It has advantages such as being non-invasive, easy to administer (with the consequent high patient compliance) and cost-effective, whilst also being easy to store and transport, and the formulations can easily be modified. However, serious drawbacks to conventional drug delivery systems (DDS) are imposed by the gastrointestinal tract (GIT). Large fluctuations in drugs’ bioavailability are found due to the influence of physiological factors such as variations in pH, high enzymatic activity as well as gastric emptying. In addition, rapid gastrointestinal transit can prevent not only the complete drug release from the dosage form, but also the full drug intake in the absorption zone (most drugs are absorbed in the stomach or the upper part of the small intestine) with the consequence of loss of dose effectiveness. This is the reason why frequent drug administrations are required to maintain the drugs therapeutic plasma level.

Gastroretentive drug delivery systems (GRDDSs) have emerged as an ideal approach to overcome these drawbacks. Their main goal is to prolong the gastric residence time (GRT) of the dosage forms in the stomach up to several hours, so that the time between dose administration is lengthened and the drug release proceeds at the desired rate [2].

Consequently, GRDDS can play a key role in:

(a) Prolonging the delivery in the stomach of particular drugs with local activity in the stomach or the upper part of the intestine (amoxicillin, for eradication of *Helicobacter pylori* in peptic ulcer diseases) [3];
(b) Slowing down the release of drugs that are soluble at acidic pH (ranitidine, H2-receptor antagonists) [4];
(c) Prolonging the release of drugs with a narrow absorption window, i.e., with low absorption in the lower part of the GIT (levodopa and carbidopa, drugs used in the treatment of Parkinson disease), and drugs with low bioavailability such as the antidiabetic metformin [5,6,7,8].

There is an extensive and valuable bibliography related to these devices [5,9,10,11,12,13,14]. Among the main types of GRDDS developed, the most promising are the low-density (floating) systems [8], which include the raft-forming formulations [9]. A brief description of the properties, requirements and drawbacks of these systems is summarized in Table 1, along with a summary of commercially available floating GRDDS for the treatment of a variety of pathologies in Table 2.

This review focuses on the polymeric materials involved in the preparation of floating gastroretentive drug delivery systems (FGRDDSs). The description of the polymers and their commercial versions with their main properties will be disclosed accordingly, as the main types of FGRDDS are described.

## 2. Single-Unit Low-Density Systems

Low-density/floating systems are the most practical and extensively studied gastroretentive dosage forms. However, the design of floating dosage forms is technically demanding. Firstly, traditional in vitro dissolution methods are not able to predict in vivo behavior with a sufficiently high accuracy [24]. Secondly, neither the European Pharmacopeia nor the American Food and Drug Administration (FDA) describe any specific methods to assess dissolution behavior and floating characteristics of these formulations. Thirdly, methods for preparing floating gastroretentive systems are often cumbersome and expensive. Finally, cost-effective large-scale production of floating drug delivery systems (FDDS) remains a challenge [25].

The benefits of low density/floating systems are rooted on the buoyancy of the dosage in the gastrointestinal fluids. Its bulk density must be lower than that found in gastric fluids (1.004–1.010 g/mL) to ensure their floatability on the gastric fluid and hence to prolong their GRT [18] (4–24 h depending on the system) [26]. A low-density system requires high fluid levels in the stomach to float and work effectively and to avoid gastric irritation. Therefore, drugs with irritant effects on the gastric mucosa are not suitable candidates for such formulations [2].

To make the formulations float in the stomach, several approaches have been developed (Table 3). One of the most common systems uses a swollen polymeric material. The gel-forming formulation entraps air, leading to systems with low density. This is the case for hydrodynamic balanced systems (HBS) and raft systems.

The density of the floating dosage forms can be reduced even further by using additives that generate a gas, such as CO_2_ once the polymer swells, making them less dense than the gastric fluids. CO_2_ is produced from an acid-base reaction in which a CO_2_ generating salt, such as bicarbonate and carbonate salts, is involved [17,27,28]. One example of an effervescent system is the reaction of citric acid and sodium bicarbonate, where the optimal stoichiometric ratio for gas generation is reported to be 0.76:1, respectively [29]. To prevent the presence of gastric acids from interfering with the CO_2_ formation, the formulations usually incorporate biocompatible acids such as tartaric acid and citric acid. Carbonate or bicarbonate may be present in the amount ranging from 5% to 50% and preferably from about 10% to 30% by weight of composition [13]. The effervescent effect, along with the swelling characteristic of hydrophilic polymers, can improve the overall floating behavior of the dosage form, i.e., floating lag time (FLT) and floating duration [27].

Although CO_2_ systems are the most prevalent, the reduction in the formulation density can also be achieved by the incorporation of air-filled chambers. For example, floating alginate beads of Ranitidine HCl, with air compartments inside to increase its residence time in the stomach, were reported [30]. Another option is the vaporization of volatile solvents confined in inflatable chambers that, once warmed into the human body can vaporize [18]. The most commonly used volatile solvents are acetone (b.p. 56 °C) [31] and dichloromethane (b.p. 39.6 °C) [32], or even mixtures of several solvents [33,34].

The incorporation of low-density materials in the formulations such as mineral and natural oils like liquid paraffin, olive oil and sunflower oil [3,35,36] have also been used. Non-ionic emulgents can also be used such as PPO-based triblock-copolymers (Poloxamer^®^ [37]) and glyceride-derivatives. Gelucire^®^ composites are examples of non-ionic emulgents, composed of mixtures of mono, di and triglycerides with PEG esters of fatty acids [38]. In addition, several glyceryl fatty acid esters have been chosen, such as glyceryl palmitostearate (Precirol^®^ ATO 5, equivalent to Gelucire^®^ 54/02) and glyceryl behenate (Compritol^®^ 888, equivalent to Gelucire^®^ 70/02). The first was used as a meltable binder and the esters of behenic acid as lipophilic diluents [6]. These compounds improve the buoyancy of the formulation (for periods even longer than 24 h), increase drug entrapment efficiency for lipophilic drugs and also impose a hydrophobic barrier towards the drug escaping from the matrices, which results in higher drug entrapment with prolonged drug release behavior [36]. Some authors have also reported the formation of floating microparticles with the use of synthetic polymers foams such as polypropylene foam powder [39] and porous polystyrene-based matrices with very low density [40].

### 2.1. Hydrodynamic Balanced Systems (HBS)

Hydrodynamic balanced systems (HBS) involve mixing the drug with a gel-forming polymer. The floatability of a non-effervescent systems relies on two possible mechanisms: the first one depends on the incorporation of a high level (20–75% *w*/*w*) of one or more gel-forming polymers that, with the drug, allows the formulation to remain buoyant over the gastric fluids [16]. When the gel-forming polymer hydrates, a gel barrier is built that controls the fluid penetration into the device and the consequent drug release from the formulation. In this system, the drug is mixed with the polymer and filled in the gelatin capsule. In effervescent formulations, a permeable outer barrier can help as the air entrapped by the swollen polymers aids the buoyancy of these dosage forms [5,20].

HBS are useful for the controlled release of drugs, which have a better solubility in gastric environments, i.e., drugs soluble at acidic pH, like the cationic molecule metformin [41]. They are also of interest for the formulations of drugs that are primarily absorbed in the stomach, such as amoxicillin [42]. However, as HBS is a matrix formulation, the release kinetics of the drug cannot be changed without changing the floating properties of the dosage form and vice versa [9].

For these systems one or more gel-forming and/or highly swellable polymers are used. One of the main types are cellulosic hydrocolloids, particularly, hydroxypropylmethyl cellulose (HPMC), which is the most commonly used for the development of non-effervescent floating systems. Other semisynthetic cellulose derivatives such as hydroxypropyl cellulose (HPC), hydroxyethyl cellulose (HEC) and sodium carboxymethyl cellulose (NaCMC) are also of interest. Another group are polysaccharides, most of them heteroglycans, for example sodium alginate (NaAlg) and the natural gums guar gum, carrageenans, gellan gum and xanthan gum [5,20].

The properties of selected semisynthetic cellulose derivatives as well as the most relevant swelling gums used in floating formulations are described below.

#### 2.1.1. Cellulose Derivatives

Cellulose type hydrocolloids are the largest group of polymers with high swelling and gelling capacities. Cellulose is the most abundant polysaccharide available worldwide and is considered an almost inexhaustible source of raw material for the increasing demand of environmentally friendly and biocompatible products.

Gelling and/or matrix forming materials for floating GRDDS must be hydrophilic and able to swell with a relevant amount of water. However, because of its highly crystalline nature with a high density of inter-chain hydrogen bonds, cellulose is not soluble or swellable in water. This highly effective spatial arrangement is facilitated by the β-1,4 linkages between glucopyranose units. Hydrogels prepared from unsubstituted cellulose have been scarcely reported because of its insolubility in aqueous solutions and in most organic solvents [38]. In order to solubilize cellulose, various substitutions have been incorporated along the glucopyranose backbone, which help break down its crystallinity. Once this 3D structure is denatured and a synthetic modification occurs, the presence of side segments prevents an organized 3D disposition from being achieved, leading to a looser material with more internal clearance. The material is then capable of absorbing a greater proportion of solvent molecules. The polymer solvation, and especially, hydration, depends on the cohesion force or the integrity of the polymer networks, which is influenced by the hydroxyl groups and size of the substituents [39].

Cellulose ethers are the most widely used polymers in GRDDS. They are semisynthetic and are prepared from natural cellulose by alkylation reactions. The chemical structures of several cellulose derivatives are recorded in Table S1 (Supplementary Information, SI). For inflatable systems, the use of the appropriate derivative is a key factor.

As can be seen, hydroxypropylmethyl cellulose (HPMC), hydroxypropyl cellulose (HPC), hydroxyethyl cellulose (HEC) and sodium carboxymethyl cellulose, (NaCMC), contain protic and/or ionic groups such as hydroxyl groups, in the case of HPMC, HPC and HEC, or carboxylate groups, as in the case of sodium CMC. As the p*K*_a_ of CMC is 4.0 [40], the ratio of ionized groups will depend on whether the treatment is conducted in fasted conditions (pH of the gastric fluid: 1.5–2.0 [20]; non ionized carboxylic acid groups predominate) or fed conditions, in which the pH can raise to values close to 6.0 [20], with the consequent ionization of the acidic groups.

- Hydroxypropylmethyl Cellulose and Methyl Cellulose (HPMC and MC)

Hydroxypropyl methylcellulose (HPMC) is the most important hydrophilic carrier material used for the preparation of oral controlled drug delivery systems (Table S1) [57]. Also known as hypromellose, HPMC belongs to the group of cellulose ethers in which one or more of the three hydroxyl groups from the cellulose glucopyranose units have been substituted forming ether linkages. It is, therefore, a semisynthetic polymer prepared from highly purified natural pulp that is etherified with the combination of methyl chloride and propylene oxide to form a water-soluble, non-ionic cellulose ether [58]. The most widely used commercialized HPMC belongs to the trade names Methocel^®^ and Pharmacoat^®^.

As HPMC is a biocompatible and viscoelastic polymer, it can be used as eye drops [58], or a controlled-delivery component in oral medicaments. An in-depth study on the role played by HPMC on the release of drugs from several pharmaceutical devices has been published by Siepmann and Peppas [57]. It was observed that HPMC had the ability to control drug release since it exhibited swelling, erosion and diffusion fronts. Displaying the possibility to formulate it with various excipients and being nontoxic and cost-effective, HPMC is a well-established material for the modulation of drug release in pharmaceutical formulations [59]. Numerous examples of its use in oral formulations can be found in the scientific literature. For example, hydrophilic polymers are often used to control the drug release rate in FGRDDS. Hydrodynamically balanced systems containing 500 mg of metformin have been prepared as a single unit floating capsule using HPMC K4M as the matrix forming polymer, PEO as the swellable additive, and ethyl cellulose (EC) as the release modifier. The best formulation demonstrated an in vitro release of 97% in 12 h in simulated gastric fluid at pH 3 and it followed zero order release kinetics. Other release modifiers such as cellulose acetate phthalate (CAP), and liquid paraffin (LP) were also tested [41]. In addition, matrices with insufficient porosity can be made to float by reducing the compaction pressure and increasing their porosity with carbon dioxide bubbles obtained from the reaction of sodium bicarbonate with the acidic dissolution medium. This was the case of floating formulations of captopril in which sodium bicarbonate in HPMC (Metolose) matrix formulation were used, so that the gastric retention time was improved by increasing the hydration of the dosage form and hence, increasing the surface area of drug diffusion [19].

- Hydroxypropyl cellulose (HPC) and hydroxyethyl cellulose (HEC)

Owing to its low Tg, HPC has been used as the main matrix-forming polymer in formulations prepared by hot-melt extrusion and 3D printing technologies, implying that the formulations could be processed at a relatively low temperature [60,61,62]. HPC has demonstrated its capacity to yield bioadhesive films [63]. The role of selected additives on the bioadhesive properties of HPC-based films was investigated and it was concluded that the incorporation of Carbomer 971P and a polycarbophil into HPC films increased bioadhesion significantly when compared to the film containing HPC and PEG 3350.

However, few examples are found in GRDDS in which HPC is the main polymeric material. Its use is usually linked to other widely used polymers such as sodium alginate and HPMC. For example, Kim et al. [26] prepared new HPMC-based gastroretentive non-effervescent tablets with floating and swelling properties for once-daily administration of pregabalin in which the amount of HPC and crospovidone (cross-linked poly(vinyl pyrrolidone), PVP) were found to be critical factors affecting in vitro dissolution and floating properties of the prepared tablets. Qi et al. [54] reported the development of ofloxacin floating tablets comprised of HPC as a compression coating agent, sodium alginate as a drug release modifier and sodium bicarbonate as an effervescent agent.

Hydroxyethyl cellulose (HEC; Table S1) is used as a gelling and thickening agent in the development of biostructures for the delivery of hydrophobic drugs. For example, mucoadhesive films of enalapril were prepared with mixtures of HEC and sodium carboxymethylcellulose and showed good swelling properties and promising controlled drug release [64].

Similarly to HPC, hydroxyethyl cellulose (HEC) has been included in multicomponent polymeric matrices so that the mixture would have the gastro-retentive properties required. For example, effervescent floating tablets of pentoxifylline were successfully prepared by using sodium bicarbonate as a gas-forming agent and a mixture of HEC and sodium alginate as a polymeric matrix. The tablets could float on the surface of the dissolution medium and sustain drug release over 24 h [55]. Another example is the formulation of losartan-loaded swellable and floatable GRDDS tablets in which the appropriate combination of HEC, sodium carboxymethyl cellulose (NaCMC) and sodium bicarbonate led to tablets able to float for more than 16 h [65].

- Carboxymethyl cellulose (CMC)

Carboxymethyl cellulose (CMC; Table S1) is a semisynthetic, non-toxic, water-soluble cellulose derivative with carboxymethyl groups (-CH_2_-COOH) bound by an ether linkage to some of the hydroxyl groups of the glucopyranose repeating units from the cellulose backbone.

Due to the anionic nature of the carboxylate groups present in NaCMC, their gel-viscosity properties can be enhanced by the interaction with non-ionic hydrocolloids, such as HPMC and HEC [66]. Thus, Chen et al. [65] developed gastro-retentive tablets based on the swelling/effervescence mechanism with a combination of HEC, NaCMC and sodium bicarbonate for administering the antihypertensive drug losartan. Tablets were found to remain floating in vitro for more than 16 h. Another example was the preparation of gastroretentive HPMC-based effervescent floating tablets of propranolol hydrochloride in which the presence of NaCMC played a key role on the floatability of the final product. The higher the concentration of NaCMC, the longer the floating lag time. Furthermore, the NaCMC and Carbopol 934P were able to alter the drug release profiles and the dimensional stability of the formulations [44].

When CMC is internally crosslinked, the hydrophilic sodium croscarmellose is obtained (commercialized as Ac-Di-Sol^®^). Being a 3D structure, its solubility is scant but can absorb many times its weight in water. Due to its enhanced long-term stability, as well as its excellent water uptake and rapid swelling properties, sodium croscarmellose is used as a superdisintegrant in pharmaceutical formulations (low quantities are needed) [67], such as tablets, to assist the formulation in disintegrating in the gastrointestinal tract promptly. For example, in the preparation of floating HPMC-based minitablets of levodopa, sodium croscarmellose (Ac-Di-Sol^®^) has been chosen as the disintegrating agent and protective filler against minitablets sticking [6].

#### 2.1.2. Other Cellulose Derivatives

Cellulose acetate phthalate (CAP; Table S2) contains about 35% phthaloyl and 24% acetyl groups. Since CAP has low water solubility, its formulation into an aqueous system is challenging. The polymer is insoluble in its non-ionized form, which predominates in acid but becomes soluble as the phthalic acid groups become ionized above pH 6. It has been used as a coating agent in beads. To develop aqueous CAP coating solutions, the polymer is primarily transformed into soluble salts either with ammonium hydroxide, sodium hydroxide or triethanolamine. These solutions can be sprayed onto tablets after proper plasticization. An interesting salt forming agent for CAP is 2-amino-2-methyl-1-propanol (MAP). CAP/MAP free films showed less aging than ammoniated CAP films when stored at 40 °C. These films had high permeability to water/acid allowing water/HCl penetration into the tablet [68].

Ethyl cellulose (EC; Table S2) is the water-insoluble cellulose ether most commonly used in film coatings for pharmaceutical purposes. The EC used in the formulation of coatings invariably has a degree of substitution (i.e., the average number of hydroxyl groups substituted per glucopyranose unit) of 2.42–2.53 [69] and because of its high glass transition temperature, it must be plasticized to improve its thermal behavior and tensile properties. It is soluble in mixtures of toluene-ethanol 4:1 [69] and ethanol (for example, solutions of EC in ethanol at 10% (*w*/*w*) have been prepared [70]). Dibutyl sebacate (DBS) and Myvacet^®^ (acetylated monoglycerides) are the two most efficient plasticizers for EC-based films, the latter produced by casting from ethanol solutions [70].

Cellulose acetate butyrate (CAB; Table S2) is another polymer used for coatings. This polymer is soluble in acetone and can form a 10% solution [71]. Umamaheshwari et al. developed floating-bioadhesive multiparticulated systems (microcapsules) of acetohydroxamic acid and cholestyramine containing sodium bicarbonate as the gas forming agent. The CAB-coated microcapsules showed better buoyancy than uncoated resin particles. In vitro growth inhibition studies were performed in an isolated *H. pylori* culture. The microspheres showed better inhibition rates than plain acetohydroxamic acid [71,72].

The addition of release modifiers, such as EC, CAP and liquid paraffin (LP), to HBS for hydrophilic drugs may also be advisable, with LP also capable of reducing the bulk density of the systems [41]. The effect of the molecular weight of EC on the drug release properties of mixed films of EC and HPMC has been studied. The spherical granules have been coated with a film and the release of a water-soluble model drug substance measured. Drug release was found to decrease with increasing EC molecular weight and these results have been correlated with the mechanical properties of the films prepared [69].

Microcrystalline cellulose (MCC; Table S2) is a plastically deforming polymer with good mechanical properties (e.g., tensile strength and porosity) under compression [53]. In native cellulose, the linear chains of polymer are bundled together like spiral microfibril on the walls of plant cells. Each microfibril exhibits a high degree of three-dimensional internal bonding resulting in a crystalline structure that is insoluble in water and resistant to reagents. There are, however, relatively weak segments corresponding to amorphous regions. Different processes such as enzyme and/or acid mediated hydrolysis destroy the amorphous regions, leaving the crystalline domains. MCC is a partially depolymerized cellulose synthesized from α-cellulose precursor in which the crystalline regions are isolated. The degree of polymerization is typically less than 400.

MCC is a valuable additive in pharmaceutical, food, cosmetic and other industries. It is an insoluble, swellable diluent, used to promote matrix integrity in oral formulations and is especially suitable for the direct compression process. The compressed tablets have good hardness and are easy to disintegrate. Avicel^®^ is a family of commercial products composed by mixtures of MCC and NaCMC in different proportions [73]. Products of Avicel PH models, with varying polymer compositions, particles sizes and moisture contents, are used in the pharmaceutical industry. Two Avicel PH specifications—PH 101 and PH 102, with good compressibility and adhesion, are widely used in tablets.

Swellable GRDDS needs to maintain its size, which requires diffusion as the predominant mechanism of release (Fickian). It was found that matrices containing a swellable diluent like microcrystalline cellulose (MCC) demonstrated predominantly a Fickian mechanism of release, whereas soluble diluents (lactose and mannitol) contributed to a mixed mechanism of release [74]. Conversely, in HMPC-based floating tablets in which MCC was incorporated, no significant effect on drug release was observed when the amount of MCC was increased. This is probably due to the good wettability of MCC and negligible delay of pore formation in the gel layer [46].

#### 2.1.3. Natural Gums

Apart from synthetic cellulose ethers, naturally occurring polymers have been utilized as hydrocolloids to effectively control the release of drugs from swellable systems [66]. Natural polymers possess favorable properties, such as biocompatibility and safety, and hence, valuable applications in the pharmaceutical and biomedical fields [56].

Natural gums—gellan gum, guar gum, carrageenans and xanthan gum—other polysaccharides, such as alginates and chitosan [55], and natural polymers such as pectin and gelatin [18] are among natural hydrocolloids or gel-forming agents that have the ability to swell in contact with gastric fluid, maintain a relative integrity of shape and have a bulk density less than the gastric content. The most commonly used natural gums (Table S3) are heteroglycans of high molecular weights. They are either water soluble or swellable, and hence, able to give viscous solutions or jelly structures [75]. Natural gums are among the most popular hydrophilic polymers because of their cost-effectiveness and regulatory acceptance. In gum-based systems, the key element to drug release from swellable polymers is the use of polymers that will undergo transition from the glassy to the rubbery state, which is characterized by a gel-like layer, on hydration by water. This transition should occur fairly rapidly so that the drug has to pass through the viscous gel layer to be released [66].

- Guar gum

Guar gum (Table S3) is a polysaccharide found in the seeds of the plant *Cymopsis tetragonolobus* (family Leguminosae). It swells rapidly in the presence of water with a translucent suspension due to the dual content of guar gum: a water-soluble fraction (ca. 85%) called Guaran and an insoluble part. Guaran is a high molecular weight hydrocolloid polysaccharide composed of 2 units of D-galactose per unit of D-mannose. Guar gum is compatible with most other plant hydrocolloids but incompatible with acetone, ethanol (95%), tannins, strong acids and alkalis. Due to its mannose units, the addition of borate ions to hydrated guar gum produces cohesive structural gels [18]. Guar gum has the ability to increase viscosity and, used in solid dosage forms, will act as a disintegrant and binder in pharmaceutical industries [76].

Navaneetha and Venkateswara [51] prepared Atorvastatin calcium floating tablets based on synthetic and natural polymers, with guar gum among them. Natural gums, compared to HPMC, displayed more predictable release retardant effects and controlled drug release profiles. They found that the tablets prepared by using guar gum were retained in the stomach for 12 h, enhancing its bioavailability.

- Carrageenans

Trade names: Gelcarin^®^, SeaSpen^®^ and Viscarin^®^. Carrageenans (Table S3) are high molecular weight, gel-forming, anionic polysaccharides extracted from red seaweeds of the Rhodophyceae class. They are sulphated linear polysaccharides of D-galactose and 3,6-anhydro-D-galactose and are comprised of three types of families depending on the number of sulfate groups: lambda (λ-carrageenan, tri-sulfate), which gives viscous solutions but does not gel; kappa (κ-carrageenan, disulfate) and iota (ι-carrageenan, monosulfate) [43]. Kappa and iota carrageenans do not dissolve in water, but form gels. They are widely used in the food industry due to their superb physical functional properties, gelling, thickening and stabilizing properties. Due to their high robustness, good compatibility and persistent viscoelasticity of the tablet during granulation and compression, they proved to be useful as tablet excipient agents. Hence, for sustained release formulations, carrageenans are suitable excipients [77]. It is worth mentioning here that the true density measurements of the carrageenans were found to be comparatively higher than those of the cellulose ethers (MC, HPMC, NaCMC and HPC) [66].

Since the sulfated groups containing carrageenans are anionic in nature, they tend to interact with non-ionic hydrocolloids, resulting in an increase in the gel viscosity. This is the case of a blend of 1:2 ratio of Viscarin (λ-carrageenan) and HPMC, where a synergistic effect on gel viscosity explains the better control these polymers have on the release of ibuprofen [66]. Although matrices based on carrageenans alone have limited use for the preparation of floating systems, carrageenans could be useful as additives because they promote water uptake by polymeric hydrophilic matrices. This is due to the high mobility of the water molecules between polymer chains wherein the sulfate groups get hydrated. Jachowicz and co-workers have demonstrated this effect for carrageenans and HPMC in HBS containing L-DOPA as a model drug [43].

- Gellan Gum

Gellan gum (Table S3) is an anionic deacetylated polysaccharide derived from the exocellular polysaccharide secreted by *Pseudomonas elodea*, a tetrasaccharide repeating unit of α-L-rhamnose, one β-D-glucuronic acid and two β-D-glucuronic acid residues. Gellan gum solutions tend to form three-dimensional network hydrogels by complexation with cations, such as calcium ions, and hydrogen bonding with water.

Gellan gum can be used for in-situ gel formation with the concurrence of Ca^2+^ ions as a crosslinking agent. Thus, Rao and Shelar [78] have developed oral in-situ gel formulations of Itopride hydrochloride based on gellan gum and HPMC K100M. They were capable of remaining buoyant over extended time spans once the gelation process took place. This study revealed that gellan gum and HPMC K100M ratios could significantly affect the drug release and the viscosity of the systems. This in-situ gel-formation methodology has also been followed by Jafar et al. [79], who prepared in situ gelling formulations of ibuprofen based on gellan gum, for the generation of gastric buoyant devices.

- Xanthan Gum

Xanthan gum (Table S3) is a high molecular weight extracellular polysaccharide produced by pure culture aerobic fermentation of carbohydrates. Bacterium *Xanthomonas campestris* produces xanthan gum naturally [76]. The structure proposed for this polysaccharide indicates that it can be considered a substituted cellulose (Table S1): the main chain is a β-1,4-glucan to which a trisaccharide branch, consisting of mannose-(β-1,4)-glucuronic acid-(α-1,2)-mannose, is α-1,3 linked every two glucoses. In addition, the internal mannoses are *O*-acetylated at position 6, and every two external mannoses carry a ketal pyruvate bridging C-4 and C-6 [80]. This results in a rigid helix-shaped polymer chain solution, which interacts with other xanthan gum molecules to form a network [18].

Xanthan gum is an anionic material, soluble in warm or cold water, and is insoluble in ethanol and ether. Its solutions at low concentration are highly viscous and stable in a wide range of pH (3–12) and temperatures (10–60 °C), as well as in the presence of enzymes and salts. Consequently, it can be used as a stabilizing agent, gelling agent, viscosity-increasing agent and thickening agent [76].

Being non-toxic and non-irritant, xanthan gum is used in food products, cosmetics and in topical and oral pharmaceutical formulations. Its presence is responsible for modulating the drug release from formulations at zero order kinetics [75]. For example, floating tablets of ofloxacin were prepared by direct compression. Xanthan gum and Polyox WSR 1105 were used as gelling agents for controlled release in combination with HPMC and the tablets showed buoyancy for more than 24 h [50].

### 2.2. Floating Tablets

Floating tablets are composed of swellable hydrophilic matrix forming polymers that swell in contact with gastric fluids, and the air entrapped in the hydrated polymers bestows the buoyancy [81]. Both for single- and bi-layered tablets, non-ionic hydrophilic polymers, such as HPMC, HPC and poly(ethylene oxide) (PEO), are commonly employed since they are not affected by pH [8]. HPMC is the most widely used material with low viscosity grades chosen for floating purpose [29]. Additionally, combinations of HPMC and alginate have been described [29].

Synthetic polymers are also well-established materials in the development of floating GRDDS. For example, polyacrylic acids (or carbomers, commercialized under the trade names of Carbopol and polycarbophil are among the most popular materials due to their versatility and excellent properties as described below. Polydextrose, a 3D hydrophilic, biodegradable gel-forming material formed from sucrose, sorbitol and citric acid, is another interesting example [82]. The incorporation of the hydrophilic and highly-swellable PEO is also of interest, with the higher molecular weight (4000 kDa) being more prevalent [82]. Another matrix former for sustained drug release is Kollidon^®^ SR, a poly(vinyl acetate) (PVAc) and poly(vinyl pyrrolidone) (povidone, PVP) (PVAc-PVP) mixture, which is particularly suitable for manufacturing pH-independent, sustained-release matrix tablets. PVAc produces coherent matrices even under low compression forces since it is a plastic material [83].

Non effervescent systems incorporate a high level (20–75% *w*/*w*) of one or more such swellable matrix-forming polymers [16]. Generally the floatability can be improved by the incorporation of wicking agents or swelling enhancers [84] such as the hydrophilic polymeric excipients crospovidone (polyvinylpolypyrrolidone, PVPP) or microcrystalline cellulose (MCC) [26], sodium croscarmellose (cross-linked NaCMC) [46] and sodium carboxymethyl starch [46], which can increase the swelling rate to maintain the buoyancy of the tablets.

Tablets usually combine a swellable polymer with an effervescent system. Thus, single-layer matrix tablets commonly incorporate bicarbonate salts [45] or other gas generating agents [53] to the mixture of the hydrocolloid gelling agent and the drug. Tablets can contain a polymeric coating layer that provides high water permeability and low CO_2_ effusion, so that the generated CO_2_ gas would remain in the tablet making it float in the gastric fluids [29]. Bilayer matrix tablets can contain polymers responsible for floating in one layer and the drug loaded in the other layer, so the entire unit floats and remains in the stomach. Another option for bilayer floating tablets is the presence of an inner layer with a composition equivalent to single-layer tablets and an external layer that behaves as an immediate-release layer. In this case, the latter may contain a disintegrating agent to facilitate its deconstruction [47] such as NaCMC, whose erosion properties are responsible for the disintegration of HPMC-Carbopol-based propranolol floating tablets [44]. Many of the swelling agents mentioned above can play this deconstruction role in floating tablets [26,47]. For example, the addition of the hygroscopic crosslinked crospovidone (Kollidon CL) is frequently used due to its excellent swelling characteristics that makes it useful as a disintegrant in pharmaceutical dosage forms [26]. Similarly to single-layer tablets, bilayer tablets can be coated with a flexible and water permeable, but CO_2_-retaining polymer film made of, for example, ethyl cellulose (EC), polyvinyl acetate (PVAc) or some types of Eudragit^®^ (trademark for random (meth)acrylate copolymers, such as poly(ethyl acrylate-*co*-methyl methacrylate-*co*-trimethylammonioethyl methacrylate chloride) in ratio 1:2:0.2) [85].

Floating tablets are commonly chosen for the controlled release of hydrophobic active pharmaceutical ingredients (APIs) and are the formulation of choice for potent drugs [86] since it is challenging to maintain tablet buoyancy in high-dose tablets due to their high bulk density [8]. For promoting the dissolution of hydrophobic drugs, the incorporation of a surfactant to the hydrophilic polymeric matrix could be necessary in order to reduce the crystallinity of the drugs, thereby increasing the aqueous solubility and dissolution rate of the APIs [87]. For example, a great variety of Gelucire^®^ products—poly(ethylene glycol)-based (PEG) surfactants derived from mixtures of mono, di and triglycerides with PEG esters of fatty acids—have been used [38]. Moreover, Poloxamer^®^ (407, 188) is a trademark that encompasses a group of amphiphilic tri-*block*-copolymers —poly(ethylene oxide)-*block*-poly(propylene oxide)-*block*-poly(ethylene oxide) (PEO-PPO-PEO)—with surfactant properties and hence, able to incorporate both hydrophilic and lipophilic molecules in the formulation. On the other hand, it has been reported that several factors, such as Tg of amorphous drugs and polymers, molecular mobility of drugs and drug-polymer miscibility, were related to and may significantly affect the physical stability of these amorphous solid dispersions. For example, relaxations of drug and polymer both make contributions to the physical instability of ASDs on aging [88].

The properties of selected synthetic polymers used in floating formulations are described below.

#### 2.2.1. Matrix Forming Synthetic Materials

- Crosslinked polyacrylates: Carbomers, Carbopol^®^ and Polycarbophyl (PCP)

Carbomers are synthetic, high-molecular-weight polyacrylic acids crosslinked with allyl ethers of polyalcohols, such as pentaerythritol polyallylether and polyallyl sucrose. They contain between 56% and 68% *w*/*w* carboxylic acid groups, with molecular weights of 7 × 10^5^–4 × 10^9^ Da. Carbomers are weak acids and their p*K*_a_ was reported to be 6.0 (±0.5). Carbopol^®^ polymer is a product brand name for Carbomers (Lubrizol Corporation). Polycarbophil is the USP/NF compendial name of another family of polyacrylic acids loosely cross-linked with divinyl glycol (and calcium ions) (Table S4 and Table 4) and are registered as Noveon^®^ polymers. Polycarbophil can absorb approximately 100 times its weight in water at neutral pH [89]. There are a great variety of Carbopol^®^ polymer grades, which differ in their physical structure and chemical composition, crosslink density, polymerization solvent, type of cross-linking, network electrical charge and physical appearance (Table 4) and hence, in their performance characteristics [90].

As three-dimensionally cross-linked structures, carbomers do not dissolve in aqueous solutions but are able to absorb and retain large amounts of water after ionization to form a gel. When the aqueous dispersion is neutralized with a common base, such as sodium hydroxide, the acidic polymer is converted into a salt and the interconnected polymer chains begin to hydrate and partially uncoil due to electrostatic repulsion, leading to a hydrated network system [91]. This hydration process is strongly exothermic and may result in a change in its free volume affecting the formation of the hydrogen bonding network in which water molecules act as binding bridges among carbomer chains, not allowing them to increase their relative mobility as much as expected [92]. When the polymer is neutralized by triethanolamine, the formed gels can tolerate high-alcohol concentrations.

The most commonly used varieties of Carbopol polymers in oral delivery are recorded in Table 4.

Polymer ratios of 3–30% are required when carbomers are used as controlled release polymers in matrix tablets. Hydrogels from Carbopol and polycarbophil are generally highly permeable to various drug compounds and can be tailored to “swell”, thereby releasing entrapped molecules through their network-like structures [93]. Adjusting the polymer concentration can fine-tune the drug release. In addition, they provide good binding characteristics, thus allowing formulation of matrix tablets without the addition of a tablet binder [94]. In contrast to linear polymers, higher viscosity does not result in slower drug release with carbomers. Lightly crosslinked carbomers such as Carbopol^®^ 971P NF polymer (lower viscosity) are generally more efficient in controlling drug release than highly crosslinked carbomers such as Carbopol^®^ 974P NF polymer (higher viscosity) [94].

Another relevant feature is mucoadhesion. Polycarbophil displays excellent mucoadhesive properties and is clearly less mucoadhesive in the intestinal fluid than in gastric fluid [95]. Its greatest bioadhesion, similarly to what happens to Carbopol systems, was demonstrated to occur under acidic conditions [89]. Its high swelling capacity permits the entanglement of the polymer chains with the mucus layer. In addition, the non-ionized carboxylic acid groups bind to the mucins by means of a hydrogen-bonding stabilizing force [96]. For example, the incorporation of carbomer 971P and a polycarbophil into HPC films increased bioadhesion significantly when compared to the film containing HPC and PEG 3350 [63]. However, there is a statistical difference in the adhesive strength of Carbopol^®^ 971P (2.20 ± 0.09 N/cm) and Carbopol 974P (1.65 ± 0.09 N/cm) containing films. This can be explained by the lower molecular weight of Carbopol^®^ 971P, which is similar to that of polycarbophil, compared to that of carbomer 974P. As molecular weights of polyacrylic acids decrease, bioadhesion increases. These polyacrylates with the lower molecular weights contain more -COOH groups available for hydrogen bonding, thereby producing a greater force of adhesion. Another supporting explanation is that Carbopol^®^ 971P is not as highly cross-linked as its higher molecular weight counterpart (974P), again making it similar to the polycarbophil [63].

- Poly(ethylene oxide) (PEO)

The trademark POLYOX™ (from Industrial Cellulosics, DUPONT [98]) denotes a group of water-soluble resins (WSR) based on poly(ethylene oxide)s (PEO) available in a wide range of molecular weights and specific viscosities (Table S4). They display a fast-hydrating behavior and can quickly form hydrogels. PEO can be used as hydrophilic polymers in pharmaceutical formulations due to their excellent matrix forming properties. PEO is non-toxic, highly water-soluble and swellable, insensitive to the pH of the biological environments and ease to produce. PEO of high molecular weights have been successfully applied in controlled release dosage forms, because the rate of swelling and erosion of the polymer allows the sustained release of APIs.

High molecular weight PEO exhibits a viscoelastic nature in its swollen state because it can form dense polymeric networks in aqueous environments [99]. Consequently, PEO is of interest as an additive to reinforce the mechanical properties of highly swellable and mechanically robust matrix tablets. For example, in matrices based on the mixture of crospovidone, poly(acrylic acid) and xanthan gum, the mechanical strength of the tablets improved substantially when PEO of high molecular weight (1000, 4000 or 8000 kDa) was added (PEO weight content = 20%). The mechanical strength of the tablets was found to improve by increasing the PEO molecular weight [82].

Regarding the release kinetics of hydrophobic drugs (such as β-lapachone), PEO can be used as a release retardant. Tablets containing β-lapachone and PEO of the highest molecular weights showed zero-order kinetics. It is worth noting that the higher the molecular weight of PEO, the lower the swelling indices of the tablets, probably due to the more hydrophobic and largely entangled structure, thereby delaying the diffusion of water molecules into the matrices and leading to a slower release of the drug [82].

Polyox^®^ materials usually form part of multi-polymeric matrices. For example, by means of an experimental 3^2^ model design, Thakar et al. aimed to improve the bioavailability of baclofen in floating tablets based on the swelling polymer HPMC K4M, Polyox WSR 303 as a release retardant and sodium bicarbonate as a gas generating agent [49]. The optimized floating dosage form was studied in vivo in rabbits and provided prolonged gastric residence time as well as an increase in bioavailability (2.34 times) compared to the commercial formulation. Another example is the preparation of HPMC-based floating tablets using sodium carbonate and citric acid as gas forming agents. In this case, xanthan gum and Polyox WSR 1105 were used as gelling agents, and hence, retardant agents for ofloxacin-controlled release [50]. The authors demonstrated that the floating tablets of ofloxacin prepared using gelling agents like Polyox and xanthan gum in combination with various grades of HPMC were found to be effective to sustain the drug release up to 1 day, with this latter property being dependent on polymer concentration.

The work of Shishu et al. [100] presents a floating system of 5-fluorouracil using hydrocolloids, such as hydroxypropyl methylcellulose (HPMC) and Carbopol^®^ 934P, and gas forming agents like sodium bicarbonate and citric acid. Different grades of the poly (ethylene oxide) (PEO; grades–WSR 1105, WSR 301, WSR 303, WSR 60 K and WSR N80) have been used for HBS with metformin as API. From the in vitro buoyancy studies, it was observed that PEO WSR 60K and PEO WSR 303 and HPMC K4M containing formulations showed good buoyancy, with floatation time up to 12 h in citrate phosphate buffer at pH 3.0.

However, amorphous or partially amorphous polymers can experience a phenomenon known as physical ageing [101]. They undergo volume and enthalpy relaxations during their storage below Tg due to the non-equilibrium nature. The volume relaxation results in the densification of the non-crystalline phase with the consequent changes in the physical thermal and mechanical behavior of the amorphous polymer [102]. When these polymers are used in controlled-release formulations, changes in drug release profiles can be observed. This is the case of PEO-based dosage forms. Due to its partly amorphous structure, PEO experienced structural changes linked to physical ageing. Both the hydration properties of the API and the molecular weight of the polymer influenced the effect of PEO’s physical aging and, therefore, the release properties of the drug from the matrix [103].

- Kollidon^®^ SR

Kollidon^®^ SR is a mixture of poly(vinyl acetate) (PVAc) and povidone (poly(*N*-vinyl pyrrolidone), PVP) and its main use is as a matrix retarding agent. It is particularly suitable for the manufacturing of pH-independent sustained-release matrix tablets by direct compression or hot melt extrusion. PVAc is a plastic material that produces a coherent matrix even under low compression forces. When the tablets are introduced into gastric or intestinal fluid, the water-soluble PVP is leached out to form pores through which the active ingredient slowly diffuses outwards. Kollidon^®^ SR contains no ionic groups and is therefore inert to drug substances and its sustained-release properties are unaffected by ions or salts [104].

#### 2.2.2. Synthetic Polymers Used as Excipient in Floating Formulations

This section describes the chemical structures and main properties of the plethora of synthetic materials that are extensively found in research works describing floating gastroretentive systems.

- Poly(meth)acrylates. Eudragit^®^

Eudragit is a family of co-polymers used as targeted drug release coatings. These polymers allow drugs to be formulated in enteric, protective or sustained-release formulations to prevent the breakdown of the drug until it has reached an area with adequate pH in the gastrointestinal (GI) tract. Once the drug reaches its target area of the GIT (i.e., duodenum and stomach) it will be released from the polymer matrix and absorbed. Targeted drug release is often used to prevent dissolution of a drug in an area where the pH is not adequate for absorption, or to help minimize GIT irritation [105].

Eudragit^®^ are poly(meth)acrylates whose physicochemical properties are determined by their chemical composition (Table 5 and Table S5) and are available in a wide range of different physical forms (aqueous dispersion, organic solution granules and powders). Classification of Eudragit polymers is shown in Table 5.

Some categories of the copolymers (Eudragit L, S, FS and E polymers) are soluble in digestive fluids [106] due to functional groups in their structure that are sensitive to pH. Eudragit L, S and FS are soluble when the carboxylic acid groups of their structure are ionized, i.e., over pH 5.5, pH 6.0 and pH 7.0, depending on the copolymer composition. Thus they are of use as protective coatings in enteric formulations. Conversely, Eudragit E polymers are soluble when the tertiary amine groups are protonated. This occurs at the acidic pH found in the stomach and is of interest in gastric formulations, playing a protective coating role.

The insoluble grades (Eudragit RL, RS NE) have a low ratio of permanently ionized quaternary ammonium groups (Eudragit RL and RS) that, even though they are insoluble in aqueous solutions at every pH, they are permeable in digestive fluids. The degree of permeability depends on the ratio of quaternary ammonium groups. The polymers in the insoluble category—Eudragit NE—display low permeability but, when used means that no plasticizer is required in the formulation. Eudragit RL, RS and Eudragit NE copolymers can control the drug release, a property that can be tailored by mixing Eudragit RL and RS polymers at the appropriate ratios. Eudragit^®^ NM is a neutral copolymer available as an aqueous dispersion that enables sustained release coatings without the need for plasticizer addition [106].

Physical aging also affects amorphous polymers used in pharmaceutical coating systems. This phenomenon has been shown to cause changes in the mechanical, permeability and drug release properties of polymeric films due to a densification and decrease in free volume of the polymer. A variety of techniques have been used to stabilize polymeric films and prevent aging [107]. For example, the storage temperature was critical for the stability of theophylline pellets coated with a blend of Eudragit^®^ RS 30 D and NE 30 D [108]. The selection of a plasticizer is also a key factor when formulating a polymeric coating dispersion. Studies have been conducted in which beads were coated with Eudragit^®^ RS 30 D containing 40% ibuprofen as the active ingredient and solid-state plasticizer [109]. The addition of a miscible, high glass transition polymer is another method that has been shown to stabilize drug release from sustained release coatings. One advantage of coating films that have high Tg is that minimal aging is expected because the storage temperatures are well below the Tg. This has been observed for cellulose acetate phthalate (CAP) films when the salt forming agent 2-amino-2-methyl-1-propanol (MAP) was used for the neutralization and dissolution of CAP in water. CAP/MAP free films were found to be superior to ammoniated CAP films with respect to extent of aging when stored at 40 °C [68].

- Crospovidone. Crosslinked poly(*N*-vinyl pyrrolidone) (PVPP)

Crospovidone (trademarks: Polyplasdone^®^, Kollidon CL)) is a water insoluble synthetic cross-linked PVP (Table S5). Crospovidone swells without gelling, a property that is advantageous for developing orally disintegration tablets (concentration of 2–5% *w*/*w*). When a compaction force is applied, the polymer deforms, then, upon contact with water, it absorbs and regains its normal structure, releasing an amount of energy capable to break the tablet. As the particle size increases, the intra-particular porosity increases, leading to a larger water uptake and faster disintegration. Several grades of crospovidone are available, differing in particle size distribution, bulk density and hydration capacity [110].

Being nonionic in nature, the disintegration efficiency of crospovidone is independent of the pH of the media and thus, a potentially suitable disintegrant for cationic drugs [111]. This polymer can also be used for solubility enhancement of poorly soluble drugs in the process of coevaporation. This process enables the drug adsorption onto crospovidone in the presence of a suitable solvent, and once the solvent is evaporated, a solid mixture with a faster drug dissolution rate is provided [110]. Sometimes, crospovidone is included as part of highly hydrophilic matrices, for example, in mixtures with poly(acrylic acid), and xanthan gum. It was found that these polymers, at a weight ratio of 1:1:1, displayed excellent swellable properties [82].

- Sodium croscarmellose. Cross-linked sodium carboxymethyl cellulose

Sodium croscarmellose (trade name: Ac-Di-Sol^®^) is an internally cross-linked sodium carboxymethylcellulose used as a superdisintegrant in pharmaceutical formulations (Table S5) [111]. This material is an insoluble and hydrophilic polymer with enhanced long-term stability. The cross-linking reduces water solubility while still allowing the material to swell and absorb many times its weight in water. As a result, it provides superior drug dissolution and disintegration characteristics, thus improving formulas′ subsequent bioavailability by bringing the active ingredients into better contact with bodily fluids. It is used in co-formulations of hydrophilic particulate materials [5]. This polymer is highly effective in direct compression, dry granulation and wet granulation processes [112].

- Poly(vinyl acetate) (PVAc)

Poly(vinyl acetate) (PVAc, Table S5) is a thermoplastic hydrophobic polymer, soluble in organic solvents and insoluble in water. It is rather brittle below its Tg (ca. 305 K) and very sticky above it. The emulsions of this plastic material, produced on a very large scale, are inexpensive and possess good adhesion to many porous substrates. PVAc serves as the film-forming ingredient in water-based (latex) formulations and is used as a coating polymer since it can provide flexible and water permeable coatings to tablets or other oral formulations [85]. It is also used in adhesives, as a plasticizer and a thickener in varied applications. When employed in coatings or adhesives, PVAc is often partially hydrolyzed to a water-soluble polymer known as polyvinyl alcohol.

- Gelucire^®^

Gelucire^®^ comprises of a group of poly(ethylene glycol)-based (PEG) surfactants derived from mixtures of mono, di and triglycerides with PEG esters of fatty acids (Table S5), which are widely used in pharmaceutical formulations. Varying the molecular weight of PEG and the fatty acid results in Gelucire-based surfactants with a wide range of HLB and melting point values (33–65 °C). Thus, Gelucire^®^ grades are named depending on their melting point (the first value) and the HLB (the second value). For example, Gelucire 39/01 has HLB value 1 and melting point of 39 °C.

Based on their HLB values, Gelucire can be classified into hydrophilic and hydrophobic grades (Table 6). Gelucire with HLB values lower than 6 are hydrophobic; 6–9 are water dispersible and above 9 are hydrophilic. Gelucire 50/13, 44/14, 48/16, 55/18, 35/10 and 48/09 are examples of hydrophilic grades and Gelucire 43/01, 39/01, 33/01, 50/02, 54/02 and 64/02 are examples hydrophobic grades [38].

The most common grades used in GRDDS are the hydrophobic G43/01 and G39/01 and the hydrophilic G50/13 and G44/14 and are present in multiple-unit floating systems.

Owing to their extreme hydrophobicity and low density, hydrophobic Gelucire are considered appropriate lipid carriers and drug release retardants in GRDDS such as multi-unit minitablets [113], lipid-based pellets [114] and floating granules [115,116,117]. Thus, sustained-release floating minitablets have been prepared using both G43/01 and G39/01 without the presence of gas generating agents [113]. They may also be part of lipid matrices in beads [114,118], play a role as floating assistant agents [119] and be of interest as a hydrophobic meltable binder [48,113].

G44/14 and G50/13 are the most commonly found hydrophilic Gelucire in GRDDS. They are water dispersible and display excellent surfactive power. Consequently, they are widely used to enhance the solubility and wettability of drugs [118]. Since their composition is mainly constituted by PEG esters, they are chosen as hydrophilic carriers in solid dispersions [114] and are generally used in the preparation of fast release formulations [116]. Their thermoplastic properties make them useful as meltable binder in melt/fusion processes, for example in fluidized hot melt granulation [120].

- Poloxamer. PEO-PPO-PEO triblock-copolymers

Poloxamers are nonionic triblock copolymers composed of a central hydrophobic chain of polyoxypropylene (poly(propylene oxide)) flanked by two hydrophilic chains of polyoxyethylene (poly(ethylene oxide); PEO-PPO-PEO; Table S5). They are readily soluble in aqueous, polar and nonpolar organic solvents and their aqueous solutions are very stable in the presence of acids, alkalis and metal ions. They can form thermoreversible gels at high concentrations (15–50%, room/body temperatures) that become liquid at cool temperatures.

Due to their amphiphilic structure, the polymers have surfactant properties and can be used to either increase the water solubility of hydrophobic substances or the miscibility of two substances with different hydrophobicities [82].

Amphiphilic substances can be used as foaming agents. Foam is defined as a dispersion of gas in a liquid or a solid and the presence of a foaming agent is essential for foam generation and stabilization [37]. When a foaming agent is added to water, the hydrophobic parts of the molecule arrange themselves in a way to minimize the area of contact with that protic solvent, while the hydrophilic part of the surfactant is responsible for their solubility in water. When a foaming agent is adsorbed into the air–water interface, the surface tension of water is lowered and the surface pressure is increased. Addition of some polymers, which leads to the formation of a surfactant–polymer complex through interactions between polymer and surfactant, contributes to the foam stability. For example, alginate foams were formed by stirring it in the presence of Poloxamer 188. The alginate can wind in microbubbles and stabilize the foam solution. When the foam solution was dripped into CaCl_2_ solution through a syringe, porous beads were formed. The water insoluble Ca–Alg was rapidly formed by gelation of alginic acid in the presence of calcium ions [37].

#### 2.2.3. Some Examples of Floating Tablets

HPMC, a hydrophilic gel sustained-release matrix, is present in the preparation of a variety of floating tablets (Table 3). Gastro-floating tablets of pregabalin were prepared with HPMC and the lipophilic cetyl alcohol as a floating-assistance agent [46]. Other low-density materials such as glyceryl behenate were used for comparative reasons. Qin et al. observed that tablets containing cetyl alcohol floated longer than those with glyceryl behenate because of its lower bulk density. In addition, the use of high viscosity HPMC retarded the release of pregabalin, which is soluble in the medium. The presence of stearyl alcohol further retarded the release because of the resistance of hydration induced by the lipophilic material.

Thapa et al. studied the development of gastro-retentive effervescent floating tablets composed of a hydrophilic matrix (PEO, HPMC and HPC) loaded with a high amount of the highly water-soluble drug metformin HCl [8]. Generally, stronger gel layers control the release of the drug, as well as providing mechanical integrity to the matrices. Among the polymers tested, PEO-based tablets had the lowest floating lag time and quick penetration of dissolution medium into the matrices. They showed the highest gel strength and the lowest drug release rate among the studied systems, whereas HPCM tablets had the lowest strength and the highest drug-release rates. Moreover, the viscosity grade of HPMC also had an impact on the mechanical strength of the gel layer. The high release rates found for HPC-based tablets was associated with poor hydration rates due to low polymer viscosity.

Mixtures of alginate and HPMC have also been used in floating tablets. Controlled-release floating tablets of ofloxacin were successfully formulated by employing NaHCO_3_ as a gas-forming agent, NaAlg as retarding agent and HPMC as a matrix forming polymer. The tablets could float on the surface of artificial gastric fluid for over 12 h and control the drug release for 12 h [29]. Likewise, effervescent floating tablets of pentoxifylline were successfully prepared by using sodium bicarbonate as a gas-forming agent and a mixture of HEC and sodium alginate as the polymeric matrix. The tablets could float on the surface of the dissolution medium and sustain drug release over 24 h [55].

Floating tablets of the antibiotic ciprofloxacin were prepared by direct compression using carbomer 971, HPMC, xanthan gum and crospovidone and sodium bicarbonate as a gas generating agent. The prepared formulations were able to float for more than 24 h with a really short floating lag time (less than 20 s) and prolonged drug release for 24 h [27].

Kadivar et al. have developed effervescence and swelling floating tablets of superior gastro-retentivity and in vivo efficacy for imatinib mesylate. They were prepared using HPMC K4M, with NaAlg and Carbomer 934P. From studies in New Zealand rabbits it was observed that the gastro-retentive tablets could increase the bioavailability around 1.5 times compared to the conventional tablets (Gleevec) [52].

Effervescent tablets of propranolol hydrochloride based on HPMC K15M were developed by the direct compression method with a sustainable drug release for 12 h in the stomach. NaCMC or Carbopol 934P was added to alter the drug release profile or the dimensional stability of the formulation [44].

Optimal floating tablets must feature two, often self-excluding characteristics: high porosity to promote floatation on the stomach contents, but also sufficient hardness to withstand destruction by gastric peristalsis [25]. Due to its unique properties, functionalized calcium carbonate (FCC) holds promise in the preparation of FDDS. It is a highly porous material that allows tablets to be further processed to a relative density < 1. In the preparation of paracetamol-loaded tablets with HPMC used as a binder and FCC as excipient, it was found that FCC can be compacted into tablets with high tensile strength at compressive pressures, which are much lower than those needed for the compaction of other excipients such as mannitol or microcrystalline cellulose (MCC). The porosity of FCC containing tablets subjected to a compressive pressure of 100 MPa can reach values up to 60%, whereas tablets containing cellulose or mannitol only have 20% void volume. This is of great relevance in the development of low-density tablets [53]. Eberle et al. designed caffeine floating DDS using the novel excipient FCC. Water-soluble PEO (Polyox™ WSR 301) and HPMC (Methocel^®^ K100) were selected as gelation-layer forming polymers to slow down the penetration of liquid into the tablet during dissolution. Citric acid was chosen as the effervescent excipient. The resulting floating tablets exhibited no floating lag time, thus lowering the risk of unpredictable, premature gastric emptying. The tablets displayed sufficient hardness after incubation in dissolution media and may withstand destruction due to gastric peristalsis [25].

## 3. Multi-Unit Low-Density Systems

Although the use of floating dosage forms is the most widely used method to achieve prolonged GRTs, multiple-unit systems avoid the ‘all-or-nothing’ gastric emptying nature of single-unit systems [121]. Compared to single-unit formulations, multiple-unit formulations show a more sustained release profile and unaffected overall performance due to unit failure. These features allow the co-administration of units with different release profiles, or incompatible substances, with improved safety margins compared to single-unit dosage forms [16]. Selected examples of multi-unit FGRDDS are recorded in Table 7.

Among the multiparticulate systems, beads, based mainly on alginate and pectin polysaccharides, have been widely investigated. Their properties are summarized below.

- Alginate salts

Naturally occurring polysaccharides sodium alginate (NaAlg; Table S6) and chitosan have received much attention in drug delivery systems for their excellent biocompatibility [132]. Alginate is a naturally occurring linear anionic heteropolysaccharide isolated from bacteria and brown seaweed and marine algae. Commercial alginates are extracted from *Laminaria hyperborea*, *Ascophyllum nodosum* and *Macrocystis pyrifera* [133]. This material is characterized by its relatively low cost, low toxicity, biocompatibility and biodegradability and consists of (1→4) linked β-D-mannuronate (M) and its C-5 epimer α-L-guluronate (G) residues.

Alginate, being an anionic polymer with carboxylic groups, is a good mucoadhesive agent [133]. This property has been key to the development of liposomes and alginate-based oral mucoadhesive formulations [134].

Alginates can be cross-linked with polyvalent cations (except Mg^2+^, [135]). Indeed, the ability of alginate to transform from liquid to gel simply in the presence of cations makes alginates an ideal matrix forming hydrogel-like polymer. The most common method to form alginate-based hydrogels is using the divalent cation Ca^2+^, which can crosslink alginate by simultaneously associating with carboxylic groups in the α-L-guluronic acid blocks from different alginate chains, forming an aqueous, ionically crosslinked network. Calcium alginate beads have been intensively exploited as drug carriers in gastroretentive floating systems [30]. Such crosslinking process stiffens and roughens the polymer and reduces its swelling properties. Consequently, a reduction in the permeability of different solutes is observed, hindering the release of embodied drugs in alginate matrices and hence, allowing these systems to be used as release retarding polymers [27,136].

- Pectin

Pectin is a structural anionic heteropolysaccharide found in the primary cell walls of terrestrial plants. Structurally, pectin consists mainly of D-galacturonic acid (GalA) units joined in chains by α(1→4) glycosidic linkages (Table S6). Some of the carboxylic groups of GalA are methyl esterified. In addition to the galacturonan segments, neutral sugars are also present in the main chain. For example, rhamnose is a minor component of the pectin backbone and introduces a kink into the straight chain. Side chains of various neutral sugars, such as D-galactose, L-arabinose and D-xylose, branch off from many of the rhamnose residues and the types and proportions of these neutral sugars vary with the origin of the pectin.

The most unique property of pectin is its ability to form gels. This is the feature that makes it an important ingredient in many food and pharmaceutical products. This property varies with the degree of esterification (DE) of the carboxylic groups to methyl ester groups. DE is simply the ratio of esterified GalA groups to total GalA groups. Pectins are divided into two main categories depending on their gelling properties: high methoxy pectin (HM-pectin), which is characterized by a DE above 50%, and low methoxy pectin (LM-pectin) having a DE below 50%. HM-pectin forms gels in aqueous media with sugars and acids. LM-pectin requires the presence of calcium ions (or other multivalent cations) for proper gel formation. In general terms, divalent cations form pectin-based gels except for magnesium, which, as in the case of alginates, does not form such 3D structures [137]. The mechanism of LM-pectin gelation relies on the well-know “egg-box” model [138] with the cooperative mechanism of binding of two or more chains, in this case, with the concurrence of the carboxylic acid groups from GalA units. It is worth noting that, similarly to what has been observed for other heteropolysaccharides, a major problem with pectins is the variability found in their chemical composition, which may result in poor reproducibility in delivery characteristics. This is key to explaining their different gelling abilities [137].

From an experimental point of view, LM-pectin hydrates and gels in either cold or hot water, in the presence of calcium ions (which increases viscosity and accelerates gelation). The ideal working pH range is pH 2.5 and pH 5.5, although the exact pH at which pectin will gel also depends on the concentration of calcium ions. For most applications, the concentration range used is between 0.15% and 3.1%.

### 3.1. Beads

Multiple-unit dosage forms have been shown to reduce inter- and intra-subject variability (Table 7). Among them, hollow beads, with a lower density than that of the GI fluids, were implemented. The most common approach for preparing these systems involves resin beads loaded with bicarbonate and coated with EC. The insoluble but permeable coating allows water to permeate through it, thus the carbon dioxide that is released does not escape, causing the beads to float in the stomach [20]. Floating beads can be prepared using polymers such as alginates [37], polycarbonate/dichloromethane [32], CAB/Eudragit RL100 mixture in acetone and Eudragit S100/isopropanol [31]. The floating beads are made by solvent evaporation, or by incorporating a gas-forming agent such as CaCO_3_ [126] or porous structural element [128]. However, the introduction of an organic solvent (used in the solvent-evaporation formation, or for incorporating the gas-forming agent such as CaCO_3,_ combined with glacial acetic acid [37]) can be a drawback. Other approaches and materials that have been reported include highly swellable hydrocolloids with light mineral oils, a mixture of sodium alginate and sodium bicarbonate, multiple unit floating pills that generate carbon dioxide when ingested, floating minicapsules with a core of sodium bicarbonate, lactose and PVP coated with HPMC and floating systems based on ion exchange resin technology, etc.

#### 3.1.1. Alginate Beads

Alginate beads have been developed as floating dosage forms to prolong the GRT since the 1980s and have frequently been employed as unique vehicles for gastroretentive floating systems [37] (Table 7). Benchmark research carried out by Whitehead et al. discussed a comparative gamma scintigraphic study of floating and non-floating calcium alginate multiple-unit beads in healthy human volunteers. Radio-labeled formulations were swallowed after a standard breakfast. The study demonstrated that the buoyancy of the floating beads experienced a five-to-six-fold increase compared to their non-floating counterparts [121].

Shishu and Aggarwal developed floating calcium alginate beads loaded with 5-fluorouracil. They found that those multiple unit floating systems were three times more effective at reducing gastric tumor incidence in mice than conventional tablet dosage forms [100]. Combinations of famotidine and quercetin for the treatment of peptic ulcers have been prepared in the form of freeze dried calcium alginate beads, which display floating properties for more than 8 h [122]. Furthermore, alginate gel microbeads, with pores size between 5 and 200 nm, have been demonstrated to efficiently encapsulate proteins. The diffusion of several proteins from alginate beads has been reported and, unlike small chemical drugs, the diffusion of larger proteins from the gels are dependent of their molecular weight [133].

The incorporation of other polysaccharides such as the cationic chitosan was also tested. Ishak et al. used an ionotropic gelation method for the development of chitosan-treated floating alginate beads of metronidazole for the eradication of *H. pylori* infection [139]. Whilst the successful development of floating alginate-based multiple-unit beads of ranitidine HCl, having an air compartment to increase its residence time in the stomach, as an oral drug delivery system for gastric retention is another example. The beads were composed of a calcium alginate or chitosan/alginate core, separated by an air compartment from a microporous semipermeable membrane of calcium alginate and PVA. This air-compartment multiple-unit system did not require any effervescent agents (sodium carbonate and calcium carbonate) or low density imparting agent (oils) [30].

Although Ca alginate beads can be prepared by simple and mild procedures, they suffer from some major limitations such as low drug entrapment, short floating duration, long floating lag time and burst drug release by leaching through the pores in the beads [35]. Some of these parameters can be improved by different approaches. For example, ionically crosslinking alginate and mixing it with other polymers such as neutral gums, pectin, chitosan, HPMC and Eudragit have been found to solve the problem of drug leaching [133]. Another approach is the incorporation of additives like low-density oils and effervescent agents, indeed, oil-entrapped alginate floating beads have been established as promising multiple-unit vehicles for gastroretentive drug delivery due to their simplicity in preparation [35]. For example, floating beads of amoxicillin trihydrate with entrapped sunflower oil were prepared by ionotropic gelation using NaAlg and HPMC as the matrix polymers and chitosan as the coating polymer. These were found to float for more than 24 h [3].

The preparation of multiple unit buoyant beads of calcium alginate for the gastroretentive delivery of ibuprofen was also addressed. The development of these systems involved the simultaneous use of two low-density materials: liquid paraffin and magnesium stearate [36]. A gastroretentive drug delivery system for *Brucea javanica* oil based on alginate-carrageenan beads were developed. For the formation of the floating beads, calcium carbonate was then added to the alginate–carrageenan–*Brucea javanica* oil aqueous dispersion. The *B. javanica* oil beads had a porous structure and exhibited up to 24 h of in vitro floatability with a load capacity of 45–55% and an encapsulation efficiency of 70–80%. The gelling polysaccharide carrageenan was used in a matrix system for the first time as a porogen to modify the release rate, by utilizing its property of acid hydrolysis at a pH below 3.5 [123].

#### 3.1.2. Pectin Beads

Similar approaches to the preparation of alginate-based beads have been conducted with the polysaccharide pectin (Table 7). Badve et al. formulated hollow calcium pectinate beads of diclofenac sodium for its chronopharmacological ability. The floating beads were structurally hollow spheres with a bulk density of less than 1 g/mL and a porosity of 34% [140]. Famotidine gastric floating calcium pectinate beads have been prepared by emulsion gelation. The gel beads were prepared by employing LM-pectin alone and in combination with the hydrophilic polymers Carbopol 934P, HPMC K15M and polycarbophil as release retardants. The presence of these polymers proved to be essential in achieving sustained drug release. Cod liver oil was incorporated to enhance the buoyancy of the systems and the formulations showed the ability to float over the gastric content for more than 24 h [124].

In another study, floating cinnarizine gel calcium pectinate beads were prepared. The researchers reported improved in vivo efficacy of up to 3.80 times, compared to conventional tablets in healthy human volunteers. Interestingly, the beads composed of LM-pectin (base), glyceryl monooleate (GMO) and labrafac lipophile WL 1349 (oil phase) had instant in vitro floating capacity, excellent floating properties, high-loading efficiency and zero-order release patterns. These properties suggest that they would be potentially suitable for once-a-day administration of cinnarizine [141].

Other authors have chosen zinc ions as ionic cross-linkers in pectinated beads. Thus, Mohan et al. described a delivery system in which the retention of ofloxacin could be achieved by the development of rice bran oil entrapped zinc pectinate beads prepared using LM pectin, alone and in combination with drug release retardant polymers such as gellan gum, karaya gum and xanthan gum. The incorporation of these gums into the zinc pectinate matrices increased the viscosity of the polymer matrix and congruently decreased the drug release. Rice bran oil was used to impart buoyancy to the beads [125].

#### 3.1.3. Other Approaches

The combination of alginate and pectin polysaccharides in the development of new beads has resulted in the improvement of alginate or pectin-based formulations. For example, an attempt was made to improve the dissolution profile of poorly water-soluble gliclazide by developing hollow, floating alginate beads by ionotropic gelatin using various biodegradable polymers, like low methoxyl gelatin, pectin and HPMC and calcium carbonate as the gas generating agent (Table 7). Particle sizes were in the range of 730–890 μm [86]. The incorporation efficiency of the alginate-pectin beads was higher than alginate-HPMC beads. This study suggests that the developed beads containing gliclazide could enhance drug entrapment efficiency, reduce the initial burst release and modulate the drug release. The in vivo study in mice demonstrated a significant hypoglycemic effect over a period of 12 h and 24 h, respectively, with HPMC and pectin beads [127]. Similarly, Talukder and Fassihi reported floatable multiparticulate hollow beads developed either using calcium and methoxylated pectin or calcium plus methoxylated pectin and sodium alginate. Riboflavin, tetracycline and methotrexate were used as model drugs for encapsulation. The results showed that calcium-pectinate-alginate beads released their contents at much faster rates than the calcium-pectinate beads (100% in 10 h vs. 50% in 10 h) [142].

There are other examples of synthetic or semisynthetic bead-forming polymers. For example, porous Eudragit^®^ L beads of metronidazole have been prepared using cetyl alcohol as a porogen. Eudragit^®^ L, metronidazole and cetyl alcohol were dissolved in acetone and then extruded into dichloromethane. The results showed that, after extruding, cetyl alcohol dissolved out from the beads already formed, resulting in a porous structure. Thus, the beads could float in simulated gastric fluid for more than 8 h. In addition, the augmentation of cetyl alcohol in the formulations significantly sustained the drug release while the beads remained floating. The results suggest that Eudragit^®^ L beads could be used as a carrier for intragastric floating drug delivery [128]. HPMC can also be the gel forming polymer in Levodopa-loaded multiparticulate systems. They have been successfully formulated as gastric floating minitablets by melt granulation and subsequent compression using glyceryl palmitostearate (Precirol^®^ ATO 5 = Gelucire^®^ 54/02) and glyceryl behenate (Compritol^®^ 888 = Gelucire^®^ 70/02) as a meltable binder and a lipophilic diluent, respectively. High and low viscosity grades of HPMC were chosen as the gel-forming polymer and the coating agent, respectively. The gas generating agents were sodium bicarbonate or calcium carbonate. When they were filled into gelatin capsules, no sticking was observed. Their ability to sustain drug release over an extended period, as well as their robustness under varying pH or agitation rates, were demonstrated [6].

### 3.2. Microballoons/Hollow Microspheres

Microballoons are completely hollow microspheres that consist of a cavity located inside the particle and an outer layer that contains the drug. The formulation of drug-loaded microballoons or hollow microspheres involves simple solvent evaporation or solvent diffusion methods [9]. They are made of low-density materials, characteristically free flowing powders with sizes lower than 200 μm [20], which display immediate buoyancy by entrapping oil or air. Polycarbonate [32], ethyl cellulose, Eudragit^®^, chitosan, alginate, low methoxylated pectin and agar are commonly used polymers [5,14] (Table 7). For example, an ethanol: dichloromethane solution (1:1) and an acrylic polymer are poured into an agitated aqueous solution of PVA, used as a dispersing agent, at 40 °C. The gas phase generated in the dispersed polymer droplet by the evaporation of dichloromethane forms an internal cavity in the microsphere of the polymer with the drug leading to microballoons capable of floating for more than 12 h [33]. A selected variety of examples are recorded below, demonstrating the breadth of work conducted on these systems.

Sato et al. have examined pharmacokinetic data of riboflavin in Eudragit^®^ S-100, and HPMC-based microballoons in which PVA was used as the dispersing agent. It was noticed that the larger microballoons (particle size 500–1000 μm) showed better intragastric floating properties in comparison to the smaller particles (particle size < 500 μm), an aspect that is likely to be beneficial as far as a sustained pharmacological action is concerned [143]. This correlation between microsphere size and floating properties was also found by Gupta et al. [144]. They published interesting research on the preparation of floating famotidine microspheres made of Eudragit S-100 by modified solvent evaporation. It was observed that, on augmenting the polymer ratio, the average particle size increased, and the size was also correlated with an improvement in their floatability. The authors used a hard gelatin capsule containing BaSO_4_ to administrate the floating microspheres to albino rabbits. They found, by radiographic image, that 1 h after the administration; all the microspheres were scattered in the stomach and able to prolong their GRT.

In another study, Lee et al. determined the effect of solvent composition and non-volatile oil on the floatation and release profile of model drugs (cyclosporine A, ketoprofen, piroxicam, tacrine HCl and tenoxicam) from Eudragit^®^ microspheres. The best formulation was obtained when the ratio of dichloromethane: ethanol: isopropanol was maintained at 5:6:4. The formulations containing oil had less dense and more porous channels [34]. Floating and bioadhesive Eudragit E based microspheres containing the antiurease drug acetohydroxamic acid (AHA) were prepared by a novel quasi-emulsion solvent diffusion method. The microballoons were coated with a 2% *w*/*v* solution of polycarbophil (Carbopol^®^ EX 55) by the air suspension coating method. The authors proposed that the polycarbophil-coated microspheres adhere to the mucosa so that they specifically release the drug in the mucosal cell lines [72].

Porous calcium silicate based floating microspheres of repaglinide were developed by emulsion solvent diffusion using Eudragit S as a polymer. The formulation demonstrated favorable in vitro floating and release characteristics. The incorporation of calcium silicate in the microspheres proved to be an effective method to achieve the desired release behavior and buoyancy [145].

Cellulose derivatives are also involved in some microsphere formulations. For example, hollow microspheres of theophylline with zero order release profiles have been prepared by a modified emulsion-solvent evaporation method using a mixture of CAB and Eudragit RL 100 (1:1) as polymers. The prepared formulations could remain afloat for more than 24 h [31]. Ranitidine hydrochloride loaded floating microspheres were prepared by solvent evaporation-matrix erosion using an EC and PEG blend. PEG was employed as a pore forming agent to induce buoyancy. Dispersion of the drug/polymer solution in liquid paraffin yielded discrete and spherical particles (between 45 and 106 μm). The size reduced when the percentage of PEG in the microspheres increased. The EC-based microspheres with a PEG content ranging between 20.0% and 33.3% showed floating properties [129].

The presence of biodegradable, ionizable polysaccharides such as alginate and chitosan in microparticulated systems has also been reported. Alginate microspheres of acyclovir were prepared by a single emulsion cross-linking method. A sustained in-vitro release of the API was observed over the period of 8 h [130]. Chitosan-based microspheres containing itraconazole were prepared by an ionotropic gelation procedure. In order to enhance the low solubility of itraconazole at pH 1.2, complexes of the drug with randomly methylated β-cyclodextrin, crosslinked poly(vinyl pyrrolidone) and starch were tested and dioctyl sodium sulfosuccinate and sodium tripolyphosphate were used as ionic cross-linkers. The best results were found when the drug was complexed with β-cyclodextrin, the ionic crosslinker was sodium tripolyphosphate and PEG 4000 was added to the formulation. The microspheres were studied by scanning electron microscopy (SEM) and the cross-section of the beads revealed a hollow cavity with thick walls and matrix structure with many pores, which seemed to be responsible for ensuring the zero-order releasing profile [131].

### 3.3. Multi-Unit GRDDS Developed by Hot Melt Extrusion (HME) and 3D Printing Techniques

The hot melt extrusion technique (HME) has been widely applied in the pharmaceutical industry with different dosage forms such as granules, minitablets, transdermal films and implants [146]. Compared with traditional methods, HME is a simpler process to continuously prepare sustained-release tablets. Recently, this technique has been employed to obtain floating GRDDS (Table 8) using insoluble polymers as matrix forming agents, mixed with a soluble polymer to facilitate water penetration in order to form a gel-like material to control the drug release [147]. Some specific components of the formulations, such as stearic acid [60,148] and stearyl alcohol [149] are necessary as a processing aid for HME. In addition, low Tg polymers such as HPC [60,61,62,150], Gelucire^®^ [119] and poly(vinylpyrrolidone-*random*-vinyl acetate) (PVP/VA) [150] are commonly used.

The group led by Repka has prepared floating pellets by HME from either hydrophilic [148] (HPMC K15M/Eudragit^®^ RS PO) and hydrophobic [119] (Gelucire^®^ 43/01) matrix-former polymers for controlled release of theophylline and cefuroxime, respectively. Other types of floating devices, based on hollow tubes with sealed ends, were obtained via HME from Eudragit^®^ RS and Eudragit^®^ E for metformin-controlled release. The systems displayed sustained release abilities up to 12 h. The buoyancy of the systems was found to depend mainly on the cylinder design and the inner/outer diameter ratio [149].

The extrudates obtained by HME can also be used for 3D printing for the preparation of multilayered dosage forms with special designs or applicability [147,151]. For example, the preparation of HPC-based tablets combining HME and 3D printing techniques have been reported for the controlled release of domperidone [62] and theophylline [60], showing buoyancy over 10 h.

Dumpa et al. have fabricated, by means of HME/3D printing technologies, filaments of HPC/EC, which were used for the manufacture of core–shell gastroretentive floating tablets with pulsatile delivery of theophylline (the lag time for the pulsatile release of the drug was from 30 min to 6 h) [61]. The same group has recently reported the preparation of floating tablets for the administration of cinnarizine using HPC and Kollidon^®^ PVP/VA64 (*random* copolymer of vinylpyrrolidone and vinyl acetate, ratio 60:40) as matrix forming polymers [150].

Other floating devices such as capsules have been achieved by means of 3D printing of PVA or polylactic acid (PLA) filaments (Table 8) [152,153,154]. These devices present hollow pockets that allow the systems to float over 24 h. The systems are designed in order to incorporate a conventional drug tablet or capsule inside.

## 4. Raft-Forming Systems

Raft systems mainly focus on achieving localized effects because floating rafts act as blockades between that esophagus and stomach. The formed raft can remain intact in the stomach for several hours, promoting the sustained release of the drug. Thus, they can be used for the effective management of gastric esophageal reflux disease, as well as gastrointestinal infection and disorders [13]. However, the mechanical strength of the systems is weak and can be easily disrupted by the migrating myoelectric complex (MMC). The strength of the alginate raft is dependent on several factors, including the amount of carbon dioxide generated and entrapped in the raft, the molecular properties of the alginate and the presence of aluminum or calcium in the antacid components of the formulation. Since the raft can be retained in the stomach for several hours, alginate-based raft-forming formulations can additionally provide longer-lasting relief than that from traditional antacids [14].

Raft-forming systems are another type of FGRDDS. This type of delivery system, initially as a solution form, usually contains sodium alginate (NaAlg) as an in-situ gel forming polymer along with calcium salts as crosslinker donors and/or carbonates or bicarbonates as effervescent agents [156]. When they come in contact with the gastric fluid, they swell and generate a viscous cohesive gel that contains entrapped carbon dioxide bubbles, leading to the formation of a continuous layer, termed as rafts [9]. Raft formation occurs rapidly, often within a few seconds of dosing since alginates can be cross-linked with polyvalent cation. The CO_2_ gas is generated and lowers the bulk density of the system, and as a result, the rafts float on the gastric fluid.

Alginate-based raft-forming formulations (Table 9) have been marketed worldwide for over 30 years under various brand names, including Gaviscon, which is a treatment for heartburn and indigestion including hiatus hernia and gastro esophageal reflux disease [14]. An interesting raft forming system of curcumin has been prepared using curcumin-Eudragit^®^ E PO solid dispersion. It aimed to prolong the GRT of curcumin and provide a controlled release therapy to treat gastric ulcer. NaAlg was used as a gelling polymer and calcium carbonate was present in the formulation for generating divalent Ca^2+^ ions and carbon dioxide to form a floating raft. These studies demonstrated that the new raft forming systems containing curcumin solid dispersions are promising carriers for a stomach-specific delivery of poorly soluble lipophilic compounds [157].

Pectins behave similarly to NaAlg. Thus, optimized raft forming systems for the water-soluble antiepileptic and anti-neuropathic gabapentin (GBP) were prepared from gellan gum, and low-methoxy pectin. The raft system achieved a zero-order release profile suitable for once-a-day administration. In vivo assessment was performed in rats to evaluate gastric residence of the gel formed. The increment in relative bioavailability of GBP from the optimized formula was 1.7 fold compared to commercially available Neurontin^®^ [158].

## 5. Conclusions

To date, extensive research work has been conducted on GRDDS to overcome the drawbacks associated with conventional dosage forms, with low-density formulations being the most promising systems. However, there is no single answer to best resolve the problems associated with each dosage form of the countless APIs available in the therapeutic arsenal. Each drug bears its particular needs that must be met by formulations capable of ensuring its bioavailability at therapeutic levels. Therefore, it is essential to assess gastroretentive dosage forms on a case-by-case basis. Critical quality attributes of low-density formulations include buoyancy, floating force, gel strength, in vitro drug release, swelling capacity, hydrogel porosity, tablet tensile strength, etc. From the formulation point of view, understanding polymer behavior and its role in formulations are crucial for the rational development of gastroretentive dosage forms. The choice of polymeric components in each formulation, either alone or in combination, is a key parameter to keep pushing forward the development of gastroretentive formulations. Furthermore, disadvantages associated with floating systems can be overcome by dual-working systems that are, in general terms, less affected by physiological conditions.

This review aims to aid the design of such systems through the knowledge of appropriate polymers, or by the development of new materials with tailor-made properties to provide optimal physicochemical properties and in-vitro and in-vivo performance.

## Figures and Tables

**Table 1:** Properties, requirements and drawbacks of current pharmaceutical technologies for floating gastroretentive formulations.

Current Pharmaceutical Technologies for Floating Gastroretentive Formulations			
Type	Requirements	Properties/How They Work	Drawbacks
Low-density/floating systems	- d < 1.004 g/mL [15]. - Highly swellable gel-forming polymers [16]. - The use of a gas-generating agent [17]. - The use of volatile liquids [18].	- Buoyant. Free volume into the formulation [19]. - They remain in the stomach for a prolonged period (high GRT) while the drug is released [20].	- A low density system may be associated with problems such as sticking together or being obstructed in the GIT, which could produce gastric irritation [9]. - They require high fluid levels in the stomach to float and work effectively [20]. - Drugs with irritant effects on the gastric mucosa are not suitable candidates for low-density systems [5]. - In some cases, the release kinetics of the drug cannot be changed without changing the floating properties of the dosage form and vice versa [9].
Raft-forming systems	- Floating properties [14].	- Floating and cohesive hydrogel [20]. - When they come into contact with gastric fluid, they lead to the formation of a continuous floating layer termed as rafts [14]. - They act as blockades between esophagus and stomach [13].	The mechanical strength of the systems is weak and can be easily disrupted by the MMC [13].

GIT: Gastrointestinal tract; GRT: gastric residence time; MMC: migrating myoelectric complex.

**Table 2:** Various floating gastroretentive formulations available in the market [9,10,20,21,22,23].

Technology/Delivery Systems	Brand Name	Active Pharmaceutical Ingredient	Drug Category	Manufacturing Company
Bilayer floating capsule	Cytotec^®^	Misoprostol	Gastroprotective	Pfizer, UK
HBS floating capsule	Madopar HBS^®^	Levodopa and benserzide	Anti-parkinsonian	Roche, UK
HBS floating capsule	Prolopa HBS^®^	Levodopa and benserzide hydrochloride	Anti-parkinsonian	Roche, UK
HBS floating capsule	Valrelease^®^	Diazepam	Anxiolytic	Roche, UK
Floating, swelling system (Tablet)	Inon Ace Tablets^®^	Simethicone, aluminum-magnesium salts	Antifoaming agent, antacid	Sato Pharma, Japan
Minextab Floating^®^—floating and swelling system	Metformin HCl	Metformin hydrochloride	Anti-hyperglycemic agent	Galanix, France
Minextab Floating^®^—floating and swelling system	Cefaclor LP	Cefaclor	Antibiotic	Galanix, France
Minextab Floating^®^—floating and swelling system	Tramadol LP	Tramadol	Synthetic opioid analgesic	Galanix, France
Effervescent floating system (Tablets)	Prazopress XL^®^	Prazosin hydrochloride	Alpha-1 antagonists	Sun Pharma, Japan
Effervescent floating system (Film coated tablet)	Zanocin OD^®^	Ofloxacin	Fluoroquinolone antibiotics	Ranbaxy, India
Effervescent floating system (Film coated tablet)	Riomet OD^®^	Metformin hydrochloride	Anti-hyperglycemic agent	Ranbaxy, India
Effervescent floating system (Film coated tablet)	Cifran OD^®^	Ciprofloxacin	Fluoroquinolone antibiotics	Ranbaxy, India
Colloidal gel forming floating system	Conviron^®^	Ferrous sulphate	Iron deficiency anemia	Ranbaxy, India
Raft forming system	Topalkan^®^	Alginic acid; aluminum-magnesium salts	Antacid	Pierre Fabre Medicament, France
Raft forming system	Almagate FlatCoat^®^	Aluminum-magnesium salts	Antacid	Pierre Fabre Medicament, France
Raft forming system	Liquid Gaviscon^®^	Sodium Alginate, sodium bicarbonate, calcium carbonate	Antacid (in reflux esophagitis)	Reckitt Benckiser Healthcare, UK

**Table 3:** Selected examples of single-unit low-density systems.

Formulation	Matrix Forming Polymers	Drug	Other Components	FT FLT	Sustained Release (h) Drug Release (%)	Technique	Comments	Ref.
HBS Capsule	HPMC K4M or PEO (60 K, or WSR 303, or WRS 301)	Metformin hydrochloride	EC, CAP, LP	12 h …	7–12 h (97%)	Physical blending	EC, CAP, LP: release modifiers.Best results: HPMC + EC.	[41]
HBS Capsules	HPMC (Metolose 65SH)	L-DOPA	Carrageenan	3–5 h 0 s	3–5 h (≈48–100%)	Physical blending	Carrageenan promoted water uptake.	[43]
Tablets	HPMC K15M	Propranolol hydrochloride	NaCMC or Carbopol 934P **Citric acid and NaHCO_3_	2 h – >12 h 7–26 s	>12 h (79–87%)	Direct compression	≥27.5% of HPMC was needed to maintain matrix integrity. Ionic interaction of NaCMC with drug. Carbopol 934P reduced FLT in contrast with NaCMC. Effervescent.	[44]
Tablets	HPMC K15M	Venlafaxine hydrochloride	Hydrogenated cottonseed oil, Carnauba wax Cetyl alcohol *NaHCO_3_	>24 h 24–104 s	24 h (>95%)	Melt granulation and compression	Cottonseed oil, carnauba wax, cetyl alcohol: Hydrophobic meltable materials and behave as retardant agents. Effervescent.	[45]
Tablets	HPMC (METHOCEL™ K100LV, K100M, K15M)	Pregabalin	PVPP/Croscarmellose (Na) Cetyl alcohol/Glyceryl behenate MCC	6.6 – >24 h 0.3–5.3 min	24 h (≈100%)	Wet granulation and compaction	Cetyl alcohol/Glyceryl behenate: floating-assistance agents. PVPP/Croscarmellose sodium: swelling and disintegrating agents.	[46]
Bilayer Tablets	HPMC (K4M, K15M, K100 M)	Pioglitazone (PG) Metformin hydrochloride (MH)	Layer of PG: NaCMC. Layer of MH: MCC, stearyl alcohol. *NaHCO_3_.	24 h (in vitro) 5 min	PG: 5 min (100%) MH: 12 h (> 95%)	Wet granulation and compaction	NaCMC: disintegrant agent. Stearyl alcohol: floating-assistant agent. In vivo evaluation. Effervescent.	[47]
Tablets	HPMC K4M G43/01	Famotidine	*NaHCO_3_	10 – >24 h 52 – >300 s	8–12 h (80–100%)	Melt granulation and compression	G43/01: hydrophobic meltable binder. Effervescent.	[48]
Tablets	HPMC K4M Polyox WSR 303	Baclofen	*NaHCO_3_	>12 h (in vitro) 4–5 s >6 h (in vivo)	12 h (in vitro) 2.34–2.43 times increase in bioavailability (in vivo)	Direct compression	In vivo studies. Effervescent.	[49]
Tablets	HPMC (Methocel™ K4M, K15M, K100M) Polyox™ (WSR 1105)Xanthan gum	Ofloxacin	**Citric acid and NaHCO_3_	>24 h 20–200 s	24 h (≈55–100%)	Direct compression	Polyox™ WSR 1105/Xanthan gum: gelling agents. Drug release rates retarded by HPMC with higher viscosities. Effervescent.	[50]
Tablets	HPMC (different viscosity grades)HPC (different viscosity grades)PEO WSR 303	Metformin hydrochloride	MCC *NaHCO_3_	24 h 1–60 s	6–24 h (100%)	Direct compression	HPMC, HPC, PEO: hydrophilic gel-forming polymers. NaHCO_3_: gas forming /drug retardant agent. Effervescent.	[8]
Tablets	HPMC (intragranular) PVPP (extragranular)	Pregabalin	HPC (intragranular) PEO, MCC (extragranular)	>24 h 8–21 s	24 h (≈75–100%)	Wet granulation and compaction	HPMC and PVPP were critical excipients in buoyancy and dissolution. HPMC, PVPP: release retardants. PVPP improved buoyancy.	[26]
Tablets	HPMC combined or not with a gum (Guar gum, Xanthan gum or Karaya gum)	Atorvastatin calcium	PVP (K-30) MCC	6–12 h 6–20 min	6–12 h (≈96%)	Direct compression	Combination of HPMC and guar gum enhanced bioavailability of the drug.	[51]
Tablets	HPMC K15M NaAlg	Ciprofloxacin hydrochloride	MCC *NaHCO_3_, CaCO_3_	8 – >12 h (in vitro) 8–165 s	12 h (in vitro) (≈65–100%)	Direct compression	Floating and bioadhesive properties. NaAlg or HPMC: release retardant.NaAlg shortens FLT. CaCO_3_: gas forming agent and crosslinker. Effervescent.	[27]
Tablets	HPMC K4M NaAlg Carbomer 934P	Imatinib mesylate	MCC *NaHCO_3_	18 – >24 h (in vitro) 23–119 s	24 h (≈44–76%)	Wet granulation and compaction	NaAlg, Carbomer: release retardant. In vivo studies (rabbits). Effervescent.	[52]
Tablets	HPMC Functionalized calcium carbonate (FCC)	Paracetamol	MCC, CaCO_3_, mannitol	…	…	Wet granulation and compaction	HPMC: binder for wet granulation. MCC, CaCO_3_, mannitol: for comparison on compactability with FCC-based tablets. FCC-based tablets with mechanical properties ≥ to those with MCC.	[53]
Coated Tablets	HPC (different viscosity grades)	Ofloxacin	MCC NaAlg (coating layer) *NaHCO_3_ (coating layer)	> 12 h 15–38 s	12 h (≈70–100%)	Wet granulation and compaction	Combination of HPC with different viscosity grades allowed to adjust FLT, FT and drug release. NaAlg behaved as a release retardant. Effervescent.	[54]
Tablets	HEC NaAlg	Pentoxifylline	MCC *NaHCO_3_	> 24 h 26–55 s	24 h (≈90–100%)	Wet granulation and compaction	HEC and NaAlg used as gel-forming polymers. Effervescent.	[55]
Tablets	HEC NaCMC	Losartan	*NaHCO_3_	16 h - >24 h 1–4.5 min	24 h (≈30–80%)	Direct compression	HEC imparted enhanced floating capacity. Tablets with higher NaCMC content had longer FLT. Effervescent.	[56]

*NaHCO_3_: gas forming agent, **Citric acid/NaHCO_3_: gas forming mixture. Abbreviations: CAP: Cellulose acetate phthalate; EC: Ehytl cellulose; FCC: Functionalized calcium carbonate; FLT: Floating lag time; FT: Floating time; G43/01: Gelucire^®^ 43/01; HBS: Hydrodynamic balance system; HEC: Hydroxyethyl cellulose; HPC: Hydroxypropyl cellulose; HPMC: Hydroxypropylmethyl cellulose; LP: Liquid paraffin; MCC: Microcrystalline cellulose; MH: Metformin hydrochloride; NaAlg: Sodium alginate; NaCMC: Sodium carboxymethyl cellulose; MCC: Microcrystalline cellulose compression enhancer; PEO: Polyethylen oxide; PG: Pioglitazone hydrochloride; PVP: Polyvinyl pyrrolidone; PVPP: Polyvinylpolypyrrolidone (Crospovidone).

**Table 4:** Chemical composition and properties of selected polycarbophil and Carbopol varieties.

Trade Name/Variety	United States (USP/NF) ^(a)^ (after 1 January 2006)	Viscosity, cP ^(b)^ (0.5 wt% at pH 7.5)	Molecular Weight between Adjacent Cross-Links [92]	Molecular Weight, Da [92,97]	Crosslinker Type ^(b)^	Use ^(b)^	Residual Solvent ^(b)^	Others ^(b)^
Carbopol^®^ 971P	Carbomer Homopolymer Type A ^(c)^	4000–11,000	237,600 Lightly cross-linked	1.25 × 10^6^	Allyl ethers of sucrose or pentaerythritol	Oral and topical	Ethylacetate (<0.5%)	More efficient in controlling drug release than Carbopol^®^ 974P
Carbopol^®^ 71G	Carbomer Homopolymer Type A ^(c)^	4000–11,000	237,600 Lightly cross-linked	1.25 × 10^6^	Allyl ethers of sucrose or pentaerythritol	Oral and topical	Ethylacetate (<0.5%)	Free-flowing granular form of Carbopol 971P NF
Carbopol^®^ 974P	Carbomer Homopolymer Type B ^(d)^	29,400–39,400	104,400 Densely cross-linked	3 × 10^6^	Allyl ethers of sucrose or pentaerythritol	Oral and topical	Ethylacetate (<0.5%)	Highly crosslinked polymer
Carbopol^®^ 934	Carbomer Homopolymer Type B ^(d)^	30,500–39,400	104,400 Densely cross-linked	3 × 10^6^	Allyl ethers of sucrose or pentaerythritol	Topical	Benzene (<0.2%)	^(e)^ Carbopol^®^ 5984 EP and Ultrez 10 NF polymers
Carbopol^®^ 934P	Carbomer Homopolymer Type B ^(d)^	29,400–39,400	104,400 Lightly cross-linked	3 × 10^6^	Allyl ethers of sucrose or pentaerythritol	Topical or Oral	Benzene (<100 ppb)	High purity grade of Carbopol^®^ 934 ^(e)^ Carbopol^®^ 974P
Noveon^®^ AA-1	Polycarbophil	2000–12,000	Lightly cross-linked	7 × 10^5^ [97]	Divinyl glycol	Topical or Oral	Ethyl acetate (<0.45%)	--

Carboxylic acid content for Carbopol (Assay %): 56.0–68.0%. the p*K*_a_ of the carbopols and polycarbophils was reported to be 6.0 (±0.5) [63]. The Carbomers Monograph in the European Pharmacopeia stipulates that benzene is limited to 2 ppm. ^(a)^ UPS-NF: Combination of two compendia, the United States Pharmacopeia (USP) and the National Formulary (NF) (https://www.uspnf.com/; accessed in May 2020). ^(b)^ https://www.lubrizol.com/Health/Pharmaceuticals/Excipients/Carbopol-Polymer-Products (Accessed in April 2020). ^(c)^ Carbomer Homopolymer Type A: Viscosity Specified (cP): 4000–11,000. ^(d)^ Carbomer Homopolymer Type B: Viscosity Specified (cP): 25,000–45,000. ^(e)^ Recommended non-benzene substitute products.

**Table 5:** Chemical composition and properties of selected Eudragit^®^ grades [106].

Eudragit Family	Example	Composition Monomer RatioMw	Functional Group Sensitive to pH	Anionic/Cationic/Non-Ionic Groups	Soluble in Digestive Fluids	Solubility in Aqueous Environments	Applications
**L**	L 100	MAA-MMA 1:1 125,000	Carboxylic group	Anionic	✔	Soluble at pH > 5.5	Enteric formulations Protective coating - Gastroresistance - Controlled drug release in intestine
**S**	S 100	MAA-MMA 1:2 125,000	Carboxylic group	Anionic	✔	Soluble at pH > 6.0	
**FS**	FS 30 D	MA-MMA-MAA 7:3:1 280,000	Carboxylic group	Anionic	✔	Soluble at pH > 7.0	
**E**	E 100	BMA-DMAEMA-MMA 1:2:1 47,000	Tertiary amino group	Cationic	✔	Soluble in gastric fluid at pH ≤ 5.0 Swellable and permeable at pH > 5.0	Gastric formulations Protective coatings - Moisture/light protection - Odor/taste masking
**RL**	RL 100	EA-MMA-TMAEMA 1:2:0.2 32,000	Quaternary ammonium group	Cationic	✘	Insoluble High permeability pH-independent swelling	Tailored drug release - Delay release - Sustained release
**RS**	RS 100	EA-MMA-TMAEMA 1:2:0.1 32,000	Quaternary ammonium group	Cationic	✘	Insoluble Low permeability pH-independent swelling	
**NE**	NE 30 D	EA-MMA 2:1 750,000	✘	Non-ionic	✘	Insoluble Low permeability pH-independent swelling No plasticizer required	

MAA: Methacrylic acid; MMA; methyl methacrylate; BMA: butyl methacrylate; DMAEMA: *N,N*-dimethylaminoethyl methacrylate; TMAEMA: trimethylammonioethyl methacrylate; MA: methyl acrylate; EA: ethyl acrylate.

**Table 6:** Application of surfactant based on the HLB range [38].

HLB Range	Water Solubility	Application
1–3	No dispersibility in water	Release retardants
3–6	Poor dispersibility in water	w/o Emulsifier
6–8	Milky dispersion	Wetting agent
8–10	Stable milky dispersion	o/w Emulsifier
10–13	Translucent to clear solution	Detergents
>13	Clear solution	Solubilizers

**Table 7:** Selected examples of multi-unit low-density systems.

Formulation	Matrix Forming Polymers	Drug	Other Components	FT FLT	Sustained Release (h) Drug Release (%)	Technique	Comments	Ref.
CaAlg beadsand solid dispersion	NaAlg	Famotidine Quercetin (QRT)	Eudragit^®^ RL100 PVP K30 *CaCl_2_	8 h 0 s	In vivo studies	Ionotropic gelation method	Eudragit^®^ RL100: for coating formation. PVP: to form solid dispersion of QRT.	[122]
Coated chitosan alginate beads	NaAlg Chitosan	Ranitidine hydrochloride	*CaCl_2_ CaAlg, PVA	72 h 0 s	11 h 100%	Ionotropic gelation method	With air compartment. CaAlg, PVA: formation of semipermeable coating.	[30]
Inner porous beads	NaAlg Poloxamer 188	Riboflavin	*CaCl_2_	6 h 0 s	10 h ≈65–85%	Ionotropic gelation method	Poloxamer 188: foaming agent.	[37]
CaAlg beads	NaAlg	Ibuprofen	MS LP *CaCl_2_	8 4.5 min	8 h 35%	Ionotropic gelation method	MS and LP: floating-assistance and release retardant agents.	[36]
Coated oil-entrapped Alginate beads	NaAlg Sterculia gum	Risperidone	Olive oil *CaCl_2_	8 h 3–6 min	8 h 64–83%	Ionotropic gelation method	Floating and mucoadhesive beads.	[35]
Coated oil-entrapped Alginate beads	NaAlg HPMC	Amoxicillin	Chitosan Sunflower oil *CaCl_2_	24 h 46 s	In vivo studies	Ionotropic gelation method	Floating and mucoadhesive beads Chitosan: coating polymer.	[3]
Hollow CaAlg beads	NaAlg Carrageenan	*Brucea javanica* oil	*CaCO_3_ *CaCl_2_	24 h 0 s	…	Ionotropic gelation method	Carrageenan: porogen. CaCO_3_: release of Ca ion crosslinker and gas forming agent. Effervescent.	[123]
Calcium pectinate beads	LM Pectin HPMC K15M Carbopol^®^ 934P Polycarbophil	Famotidine	Cod liver oil *CaCl_2_	24 h 0 s	8 h (80%) Biphasic release	Emulsion gelation	Cod liver oil: floating-assistance agent (20% was necessary). Other polymers behave as release retardant.	[124]
Zinc pectinate Beads	LM Pectin	Ofloxacin	Gellan Gum, Karaya Gum, Xanthan Gum Rice bran oilZnCl_2_	24 h 0 s	8 h 60–88%	Ionotropic gelation method	Gellan Gum, Karaya Gum, Xanthan Gum: Release retardants. Rice bran oil: floating-assistance agent. Zinc ions: crosslinker.	[125]
Coated CaAlg beads	NaAlg Gelatin Pectin HPMC	Gliclazide	*CaCO_3_ *CaCl_2_	10 h …	10 h At pH 1.2: 33–46% At pH 5.8: 82–95%	Ionotropic gelation method	CaCO_3_: release of Ca ion crosslinker and gas forming agent. Effervescent.	[126,127]
Minitablets (MT)	G43/01, G39/01	Nimodipine	COM PRE	14 h<1 min	12 h (95%)	Melt granulation and compression	COM: blend of esters of behenic acid with glycerol. PRE: glyceryl palmitostearate COM, PRE, G39/01, G43/01: release retardants.	[113]
Porous beads	Eudragit^®^ L	Metronidazole	Cetyl alcohol	>8 h 0 s	8 h >90%	Solvent evaporation and extrusion	Cetyl alcohol: floating-assistance agent, porogen, and release retardant. Solvent: acetone and extruded into DCM.	[128]
Lipid-based beads	G43/01 G50/13	Cinnarizine	Sterotex^®^ Pluronic^®^ F-127	>24 h 0 s	8 h (≈45–95%)	Hot melt method	Sterotex^®^: Hydrogenated cotton seed oil. Pluronic^®^ F-127 enhanced drug release.	[118]
Lipid-based pellets	HPMC K4M G44/14 G50/13	Berberinehydrochloride (BERH)	G43/01 COM MCC NaHCO_3_	5–9 h 0 s	8 h (70–99%)	Hot melt method	COM, G43/01: release retardants. MCC: spheronizing aid. NaHCO_3_: gas generating agentEffervescent.	[114]
Granules	HPC G50/13	Metronidazole	COM NaHCO_3_, citric acid PEG 8000	8–0 0 h≈40–100% …	>10 h (>60%)	Fluidized hot melt granulation	COM, G50/13: meltable binders. PEG 8000: release enhancer. G50/13 and HPC increased drug release. Citric acid + NaHCO_3_: gas generating mixture. Effervescent.	[120]
Granules	G43/01	Torsemide	…	8 h 0 s	8 h (86%)	Melt granulation	Lipid carrier: G43/01.	[115]
Microspheres	EC	Ranitidine hydrochloride	PEG 4000	4–10 h 5–10 min	4–6 h (85–100%)	Solvent evaporation- matrix erosion method	PEG: pore forming agent. PEG (20–33%) induced buoyancy. Reduction in size at higher PEG content.	[129]
Bioadhesive Microspheres	NaAlg	Acyclovir	LP Technetium-99m SnCl_2_, CaCl_2_	>4 h (in vivo) …	8 h (in vitro) (40–72%)	Emulsification phase separation method	Mucoadhesive properties. In vivo studies. (Radio-labeled microspheres). Higher sizes at high polymer conc. Ca ions: crosslinker.	[130]
Microspheres	Eudragit^®^ S-100	Famotidine	PVA	20 h 0 s	4–20 h ≈85–100%	Solvent evaporation method	Formation of O/W emulsion. PVA: emulgent. Porous formed by solvent evaporation (DCM).	[130]
Microspheres	CTS	Itraconazole	MβCD, PEG TPP DOS	12 h (in vitro) 6.5 h (in vivo)	8–12 h<40 mg of drug	Ionotropic gelation method	MβCD, PEG: drug solubilizing agents. DOS, TPP: crosslinking agents. Drug release decreased with higher CTS concentration.	[131]
Multiparticulates	For MH: G39/01, G43/01 For GLB: G50/13 + PEG	Metformin hydrochloride (MH) Glibenclamide (GLB)	For MH: EC, MC, MCC For GLB: …	MH: 10–12 h GLB: 7–11 h …	MH: 8 h (80%) GLB: 3 h (80%)	Hot melt method	G50/13: good carrier for fast release.EC, MC, MCC: granulating agents.	[116]
Microparticles	HPMC (various grades)Eudragits^®^ S100, L100, 100-55	Risperidone	G43/01, G44/14, G50/13 COM, GMS, Geleol mono and diglyceride MβCD, HPβCD	12 h …	12 h (71–93%)	Emulsion solvent diffusion technique	Lipid carriers: GMS; Geleol mono and diglyceride; G43/01, COM. MβCD, HPβCD: drug solubility and stability enhancers. Best polymers: Eudragit^®^ S100, HPMC E50.	[117]

*Ca ions: crosslinker. Abbreviations: CaAlg: Calcium Alginate; CNZ: Cinnarizine; COM: Compritol 888 ATO; CTS: Chitosan; DCM: Dichloromethane; DOS: Dioctyl sodium sulfosuccinate; EC: Ehytl cellulose; FLT: Floating lag time; FT: Floating time; G39/01: Gelucire^®^ 39/01; G43/01: Gelucire^®^ 43/01; G44/14: Gelucire^®^ 44/14; G50/13: Gelucire^®^ 50/13; GLB: Glibenclamide; GMS: glyceryl monostearate; HPC: Hydroxypropyl cellulose; HPMC: Hydroxypropylmethyl cellulose; HPβCD: Hydroxypropyl-betacyclodextrin; LP: Liquid paraffin; LMP: Low methoxylated pectin; MβCD: Methyl-betacyclodextrin; MC: Methyl cellulose; MCC: Microcrystalline cellulose; MH: Metformin hydrochloride; MT: Minitablets; NaAlg: Sodium alginate; PEG: Polyethylene glycol; PRE: Precirol ATO05; PVA: Polyvinyl alcohol; PVP: Polyvinyl pyrrolidone; TPP: Sodium tripolyphosphate.

**Table 8:** Selected examples of floating gastroretentive drug delivery systems (GRDDSs) prepared by hot melt extrusion and/or 3D printing.

Formulation	Matrix Forming Polymers	Drug	Other Components	FT FLT	Sustained Release (h) Drug Release (%)	Technique	Comments	Ref.
Pellets	Eudragit^®^ RSPO HPMC K15M	Theophylline	Stearic acid	24 h 0 s	12– 18 h (89–96%)	HME	Eudragit^®^ RSPO: insoluble matrix former. HPMC K15M: for control release. Ethanol: foaming agent. Stearic acid: processing aid.	[148]
Lipid-based granules	G43/01	Cefuroxime Axetil	Kolliphor^®^ TPGSG44/14	8–12 h 0 s	12 h (≈20–80%)	HME	G43/01 imparts buoyancy. Kolliphor^®^ TPGS and G44/14 behave as release enhancers. Best release profiles with G43/01 and Kolliphor^®^ TPGS.	[119]
Hollow tubes	Eudragit^®^ RSPO Eudragit^®^ E PO	Metformin	Stearyl alcohol	12 h …	4–12 h (98%)	HME	Sealed hollow tubes with inherent buoyancy. Stearyl alcohol: plasticizer.	[149]
Tablets	HPC	Domperidone (DOM)	BaSO_4_	10 h (in vitro) 8–10 h (in vivo, rabbits) …	12 h (in vitro) (99%)	HME and 3D printing	HPC/DOM filaments obtained by HME for 3D printing into tablets. BaSO_4_: label for in vivo studies.	[62]
Tablets	HPC	Theophylline (THEO)	Stearic acid	10 h 0 s	8 h (90%)	HME and 3D printing	HPC/THEO filaments obtained by HME for 3D printing into tablets. Stearic acid: processing aid.	[60]
Capsular devices	HPC EC	Theophylline	For core tablet: Croscarmellose sodium, MCC, Mg stearate	1.5–6 h 0 s	Pulse release with lag time from 0.5 to 6 h	HME and 3D printing	Core tablet (obtained by compression) into a sealed 3D-printed tablet. Pulsatile DDS with controlled lag time. EC showed significant effect on the lag time (at EC 0.5%, lag time 6 h).	[61]
Tablets	HPCPVP/VA 64	Cinnarizine	…	6–12 h 0 s	6–12 h (80%)	HME and 3D printing	PVP/VA 64 improved extrudate physical properties, leading to printable filaments.	[150]
Tablets	HPMC K4MHPMC E15	Dypiridamole	MCCPVP K30	>12 h 0 s	12 h (≈40–90%)	3D printing	Extrusion-based 3D printing technology. HPMC E15 and PVP: binder agents. MCC: extrusion molding agent.	[155]
Capsular devices	PLA filaments	Acyclovir	For drug tablets: HPMC, MCCBaSO_4_	24 h in vitroLGT: 1 min12 h in vivo (beagle dogs) …	3 h (in vitro) (80%)	3D printing	Acyclovir tablet inside the floating device. BaSO_4_: label for in vivo studies.	[153]
Capsular devices	PLA filaments	Baclofen tablets	…	24 h 0 s	2–6 h (80%)	3D printing	Immediate release drug tablet inside the device.	[154]
Capsular devices	PVA filaments	Amoxicillin capsules	BaSO_4_	14 h in vitro 0 s 10 h in vivo (rabbits)	90–180 min (in vitro) (≈80–100%)	3D printing and thermal crosslinking	Conventional amoxicillin capsule inside the device. FT increased after crosslinking. BaSO_4_: label for in vivo studies.	[152]

Abbreviations: DDS: Drug delivery system; DOM: Domperidone; EC: Ehytl cellulose; FLT: Floating lag time; FT: Floating time; G43/01: Gelucire^®^ 43/01; G44/14: Gelicire^®^ 44/14; HME: Hot-melt extrusion; HPC: Hydroxypropyl cellulose; HPMC: Hydroxypropylmethyl cellulose; MCC: Microcrystalline cellulose; PLA: Polylactic acid; PVA: Polyvinyl alcohol; PVP: Polyvinyl pyrrolidone; THEO: Theophylline.

**Table 9:** Selected examples of raft systems prepared by ionotropic gelation.

Matrix Forming Polymers	Drug	Other Components	FT FLT	Sustained Release (h) Drug Release (%)	Comments	Ref.
NaAlg	Curcumin	Eudragit^®^ (R) EPO **CaCO_3_, *CaCl_2_	>24 h 3–76 s	8 h 60–85%	Eudragit^®^ (R) EPO: drug carrier. Effervescent.	[157]
NaAlg HPMC K100M	Mebeverine HCl (MbH)	**CaCO_3_ COM, PRE	>12 h 15–25 s	5 h 100%	Higher Alg conc. retarded drug release. Lipids: PRE, COM. Floating-assistant agents. In vivo studies. Effervescent.	[159]
NaAlg Gellan gum (GG)	Metronidazole	**CaCO_3_ COM, PRE, GMS	>24 h 1 min	4–6 h 75–90%	Lipids: GMS, PRE, COM. Floating-assistant agents. Ca ions: crosslinker of Alg and GG. GG ↓ gelation capacity. A modification in formulation was needed. Effervescent.	[160]
NaAlg LM-pectin	…	***Citric acid + NaHCO_3_ **CaCO_3_	> 8 h 50 s	…	Ca ions: crosslinker of Alg and pectin. Effervescent.	[161]
LM-pectin Gellan gum	Gabapentin	GMOEudragit^®^ NE 30D *CaCl_2_	> 24 h 0 s	8 h 55–100%	Ca ions: crosslinker of pectin and GG. GMO: floating-assistant agent and drug release retardant. Eudragit^®^ NE 30D: to coat the drug.	[158]
Gellan gum HPMC (K100M)	Itopride HCl	**CaCO_3_	>12 h 76–98 s	10–12 h (>95%)	HPMC: drug release retardant. In vivo studies. Effervescent.	[78]

*Ca ions: crosslinker; **CaCO_3_: release of Ca ion crosslinker and gas forming agent; ***Citric acid + NaHCO_3_: gas-generating mixture. Abbreviations: COM: Compritol 888 ATO; FLT: Floating lag time; FT: Floating time; GG: Gellan gum; GMO: Glyceryl monooleate; GMS: Glyceryl monostearate; HPMC: Hydroxypropylmethyl cellulose; LM-pectin: Low methoxylated pectin; MbH: Mebeverine HCl; NaAlg: Sodium alginate; PRE: Precirol ATO05.

## Supplementary Materials

The following are available online at https://www.mdpi.com/1999-4923/12/7/636/s1, Figure S1. Chemical composition of Methocel products and different types of HPMC (USP) classified according to their degree of methoxy- and hydroxypropoxy-substitution. Table S1. Semisynthetic cellulose derivatives. Chemical structures and tradenames. Table S2. Other semisynthetic cellulose derivatives. Chemical structures and tradenames. Table S3. Swelling, gelling and matrix forming materials used for floating GRDDS. Natural gums. Table S4. Matrix forming materials useful in floating tablets. Synthetic polymers. Table S5. Selected polymeric excipients useful in floating tablets. Synthetic polymers. Table S6. Swelling, gelling and matrix forming materials used for floating GRDDS. Alginates and pectins. Table S7. USP specifications for different types of HPMC, classified according to their degree of methoxy- and hydroxypropoxy-substitution and their equivalency with Methocel^®^ produts. Table S8. Properties of various Methocel Cellulose Ethers. Table S9. Chemical composition and properties of Poloxamer grades [14].

## Abbreviations

APIs	Active pharmaceutical ingredients
Ac-Di-Sol^®^	Sodium croscarmellose
AHA	Acetohydroxamic acid
BMA	Butyl methacrylate
CaAlg	Calcium Alginate
CAB	Cellulose acetate butyrate
CAP	Cellulose acetate phthalate
CMC	Carboxymethyl cellulose
CNZ	Cinnarizine
COM	Compritol 888 ATO
CTS	Chitosan
DBS	Dibutyl sebacate
DCM	Dichloromethane
DDS	Drug delivery system
DE	Degree of esterification
DL	Drug loading
DMAEMA	2-(*N,N*-dimethylamino)ethyl methacrylate
DOM	Domperidone DOS
EA	Ethyl acrylate
EC	Ethyl cellulose
EE	Encapsulation efficiency
FCC	Functionalized calcium carbonate
FDA	American Food and Drug Administration
FGRDDS	Floating gastroretentive drug delivery system
FLT	Floating lag time
FT	Floating time
G39/01	Gelucire^®^ 39/01
G43/01	Gelucire^®^ 43/01
G44/14	Gelucire^®^ 44/14
G50/13	Gelucire^®^ 50/13
GalA	D-galacturonic acid
GIT	Gastrointestinal tract
GLB	Glibenclamide
GMO	Glyceryl monooleate
GMS	Glyceryl monostearate
GRDDS	Gastroretentive Drug Delivery System
GRT	Gastric residence time
HBS	Hydrodynamic balance system
HEC	Hydroxyethyl cellulose
HME	Hot-melt extrusion
HM-pectin	High methoxy pectin
HPC	Hydroxypropyl cellulose
HPMC	Hydroxypropylmethyl cellulose
HPβCD	Hydroxypropyl-β-cyclodextrin
LCST	Lower critical solution temperature
LMP	Low methoxylated pectin
LM-pectin	Low methoxy pectin
LP	Liquid paraffin
MA	Methyl acrylate
MAA	Methacrylic acid
MAP	2-amino-2-methyl-1-propanol
MB	Microballoon
MC	Methyl cellulose
MCC	Microcrystalline cellulose
MH	Metformin hydrochloride
MMA	Methyl methacrylate
MMC	Migrating Myoelectric Complex
MO	Mineral oil
MT	Minitablets
MTS	Magnesium trisilicate
MβCD	Methyl-β-cyclodextrin
NaAlg	Sodium alginate
NaCMC	Sodium carboxymethyl cellulose
PAA	Poly(acrylic acid)
PEG	Polyethylene glycol
PEO	Polyethylene oxide
PG	Pioglitazone hydrochloride
PLA	Polylactic acid
PPO	Polypropylene oxide
PRE	Precirol^®^ ATO05
PVA	Polyvinyl alcohol
PVAc	Poly(vinyl acetate)
PVP	Polyvinyl pyrrolidone
PVP/VA	Poly(vinylpyrrolidone-co-vinyl acetate)
PVPP	Polyvinylpolypyrrolidone (Crospovidone)
SI	Supplementary Information
Tg	Glass transition temperature
THEO	Theophylline
TMAEMA	Trimethylammonioethyl methacrylate
TPP	Sodium tripolyphosphate
WSR	Water-soluble resins
XrL	Crosslinking
