# Supplementary material for: In-Depth Study into Polymeric Materials in Low-Density Gastroretentive Formulations
Source: Pharmaceutics. 2020 Jul 7;12(7):636. doi: 10.3390/pharmaceutics12070636 (PMC7408198; doi:10.3390/pharmaceutics12070636)
Supplement: Supplementary file 1 [file pharmaceutics-12-00636-s001.pdf]

pharmaceutics

Supplementary Materials: In-Depth Study into Polymeric Materials in Low-Density Gastroretentive Formulations

Nieves Iglesias, Elsa Galbis, Lucía Romero-Azogil, Elena Benito, Ricardo Lucas, M. Gracia García-Martín and M.-Violante de-Paz*

1. Tables with the chemical structure of the polymers most commonly used in FGRDDS

Table S1. Semisynthetic cellulose derivatives. Chemical structures and tradenames.

Swelling, gelling and matrix forming cellulose derivatives			
**Material**	**Acronym and common names /Tradename**	**Substituents in glucopyranose units** 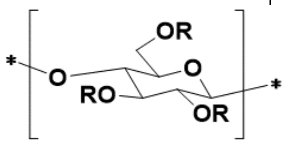	**Molecular Weight (kDa)**
Hydroxypropylmethyl cellulose/ Methyl cellulose	HPMC/MC Hypromellose/ Methocel®, Metolose®	R = $-CH_2CH(OH)CH_3$ or $-CH_3$ or -H; R = $-CH_3$ or -H	30-1,200
Hydroxypropyl cellulose	HPC/ Klucel ® HF	R = $-CH_2CH(OH)CH_3$ or -H	40-1,150
Hydroxyethyl cellulose	HEC	R = $-CH_2CH_2OH$ or -H	90-1,300
Carboxymethyl cellulose (and its sodium salts)[(a)] Sodium croscarmellose[(b)]	CMC/NaCMC XrL-CMC/ Ac-Di-Sol®	R = $-CH_2COONa$ or $-CH_2COOH$ or -H	90-700
[(a)]CMC pK_a = 4.0; [(b)]Cross-linked CMC; tradename: Ac-Di-Sol®			

Table S2. Other semisynthetic cellulose derivatives. Chemical structures and tradenames.

Other cellulose derivatives		
**Material**	**Acronym and common names/ Tradename**	**Substituents in glucopyranose units** 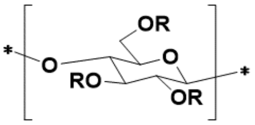
Cellulose acetate phthalate	CAP	R = $-COCH_3$ or $-CO-C_6H_4-COOH$ or -H
Ethyl cellulose	EC	R = $-CH_2CH_3$ or -H
Cellulose acetate butyrate	CAB	R = $-COCH_3$ or $-COCH_2CH_2CH_3$ or -H
Microcrystalline cellulose (mixtures of MCC and NaCMC)	MCC Avicel®	R = -H

Table S3. Swelling, gelling and matrix forming materials used for floating GRDDS. Natural gums.

Heteroglycans	
**Material**	**Chemical structure**
Guaran<br>(From Guar Gum)<br>Nonionic polysacch.	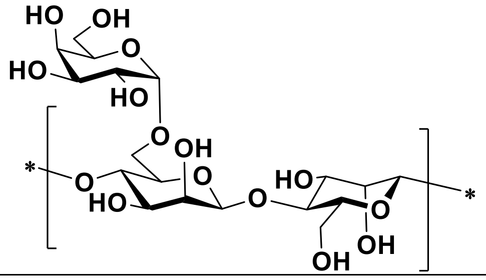
Carrageenans:<br>kappa: R = -H<br>Iota: R = $-SO_3^-$<br>Anionic polysacch.	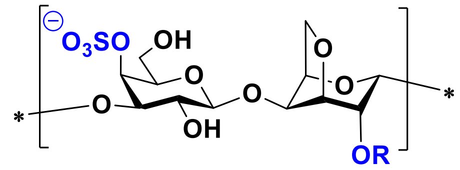
Gellan gum<br>Anionic polysacch.	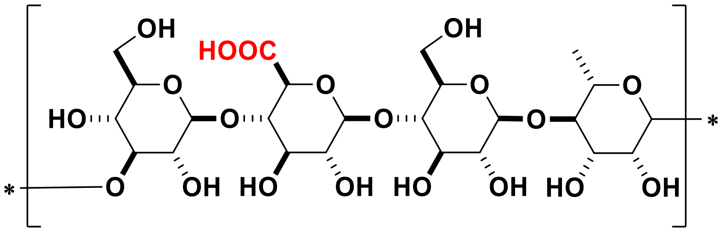
Xanthan gum<br>Anionic polysacch.	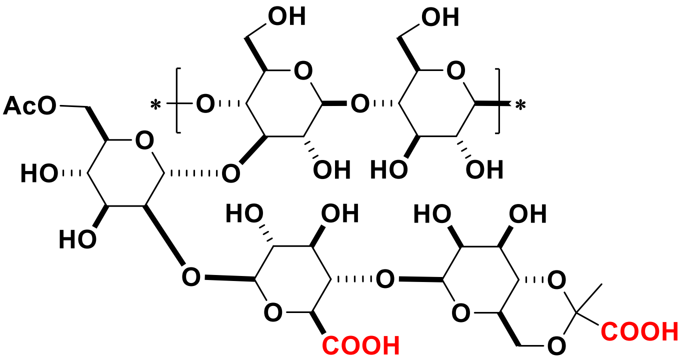

Table S4. Matrix forming materials useful in floating tablets. Synthetic polymers.

**Material**	**Acronym / Tradename**	**Chemical structure**
Poly(acrylic acid)<br>crosslinked<br>(Carbomer)<br>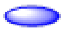 Crosslinkers	XrL PAA /<br>Carbopol®<br>Noveon®	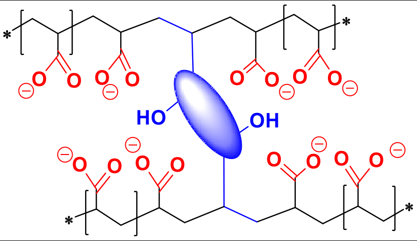
Poly(ethylene oxide)	PEO<br>Polyox®,<br>Carbowax® [1]	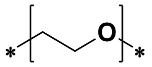
Mixtures of<br>poly(vinyl acetate) and poly(N-vinyl pyrrolidone)	PVAc + PVP<br>Kollidon® SR	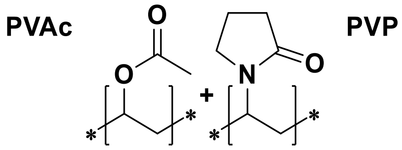

Table S5. Selected polymeric excipients useful in floating tablets. Synthetic polymers.

Synthetic polymers used as excipients		
**Material**	**Acronym / Tradename**	**Chemical structure / uses**
Poly(meth)acrylates	Eudragit	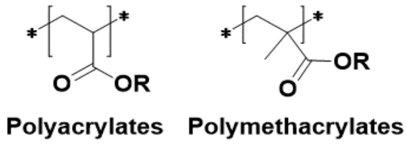<br>Protective coatings<br>Sustained drug release
Poly(*N*-vinyl pyrrolidone)<br>Crosslinked<br>Crospovidone	PVPP /<br>Polyplasdone®	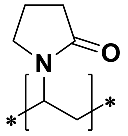<br>- Superdisintegrant;<br>- Solubility enhancer for drugs
Sodium carboxymethyl cellulose<br>Crosslinked<br>Croscarmellose	Xr-NaCMC /<br>Ac-Di-Sol®	- Superdisintegrant;<br>- Co-formulation of hydrophilic particulate material
Poly(vinyl acetate)	PVAc	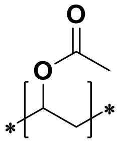<br>- Plasticizer<br>- Film-forming material<br>- Coating polymer
Mixtures of PEG esters of fatty acids with mono-, di- and triglycerides	Gelucire®	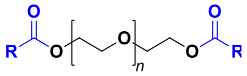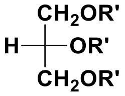<br>R = long aliphatic chain; R': -H or -CO-Aliph. chain<br>Surfactants
Poly(ethylene oxide)-*block*-poly(propylene oxide)- *block*-poly(ethylene oxide)	PEO-PPO-PEO<br>Poloxamer ®	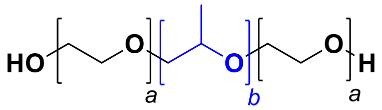<br>- Surfactant<br>- Foaming agent

Table S6. Swelling, gelling and matrix forming materials used for floating GRDDS. Alginates and pectins.

**Material**	**Chemical structure**
Alginic acid and alginate salts [sodium (NaAlg) and Calcium]<br>Anionic polysacch.	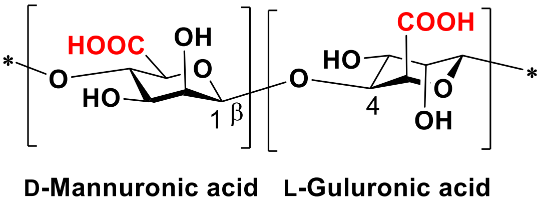
Pectin<br>Ratio (R: -CH3/R: -H)<br>≥ 50: HM-pectin<br>< 50: LM-pectin	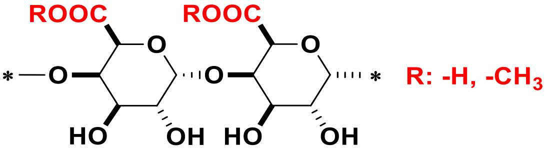

2. Additional information on selected polymers

2.1. Hydroxypropylmethyl Cellulose and Methyl Cellulose (HPMC and MC)

HPMC is available with various substitution types of hydroxypropoxy and methoxy content as well as in a wide range of molecular weights and viscosity grades. All these parameters have a strong influence in their physicochemical properties, such as swelling, hydration rates and release kinetics when they are the main component in pharmaceutical matrices. For example, swelling is enhanced for higher molecular weight HPMC grades [2]. The methoxy group content can vary between 16.5% and 30% and the hydroxypropoxy group content ranges from 4 to 32 %. The USP distinguishes four different types of HPMC, classified according to their relative $–OCH_3$ and $–OCH_2CH(OH)CH_3$ content: HPMC 1828, HPMC 2208, HPMC 2906 and HPMC 2910. The first two numbers indicate the percentage of methoxy-groups, the last two numbers the percentage of hydroxypropoxy-groups. The exact limits for the degree of substitution defining the respective HPMC types are given in Table S7 and can be visualized in Figure S1 [3]. HPMC possesses increased organo-solubility and thermo-plasticity compared to other methyl cellulose counterparts.

METHOCEL® brand cellulose ethers (METHOCEL is the trademark of The Dow Chemical Company) are methylcelluloses (MC) or HPMC with various molecular weights and substitution levels. The first letter of the product designation specifies the chemistry of the cellulose ether. "A" indicates all methylcellulose products; "E," "F," "K," and "J" indicate hydroxypropylmethyl cellulose products of different substitution levels (Table S8, Figure S1). The number that follows a letter specifies the viscosity of a 2% aqueous solution [4].

Table S7. USP specifications for different types of HPMC, classified according to their degree of methoxy- and hydroxypropoxy-substitution and their equivalency with Methocel® produts**.**

**Substitution type**	**Methoxy ($-OCH_3$, %)**		**Hydroxypropoxy ($-OCH_2CH(OH)CH_3$ %)**		**Molecular Weight (kDa)**[5]	**Methocel product**
	**Min.**	**Max.**	**Min.**	**Max.**		
HPMC 1828	**16.5**	20.0	23.0	32.0	-	J
HPMC 2208	19.0	24.0	4.0	12.0	164-1,200	K
HPMC 2906	27.0	30.0	4.0	7.5		F
HPMC 2910	28.0	30.0	7.0	12.0	400-746	E

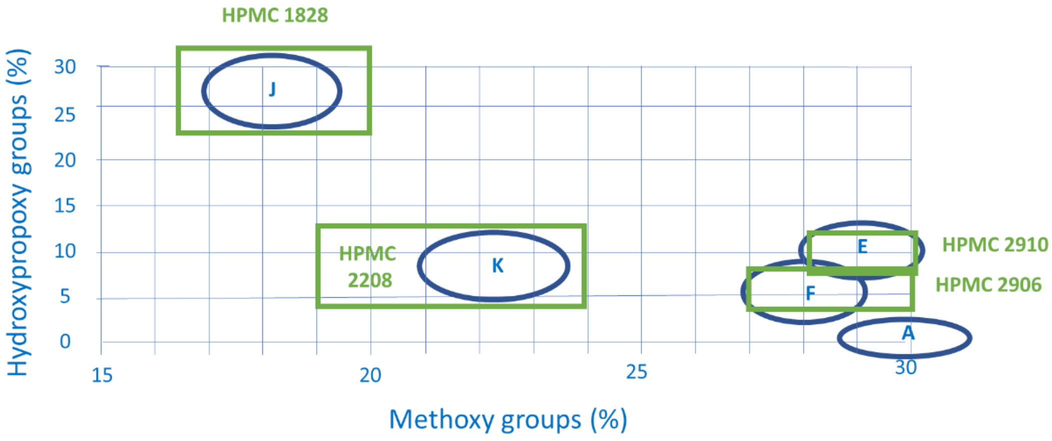

Figure S1. Chemical composition of Methocel products and different types of HPMC (USP) classified according to their degree of methoxy- and hydroxypropoxy-substitution.

Table S8. Properties of various Methocel Cellulose Ethers.

**Product**	Methoxy ($-OCH_3$, %)	Hydroxypropoxy ($-OCH_2CH(OH)CH_3$ %)	**Viscosity (mPa•s) (2% w/w in water)**	**Gel Temperature (°C)**
METHOCEL K100	23.0	**6.5**	80 - 120	70
METHOCEL F50	28.5	6.0	40 - 60	56
METHOCEL E50	29.0	11.5	45 - 55	57
METHOCEL 181(E15)	29.0	10.0	13 - 18	57
METHOCEL A15 LV	30.0	—	12 - 18	48

Even though hydrophobic groups such as methyl or hydroxypropyl moieties are introduced at the cellulose backbone, the polymer retains enough hydrophilicity [with hydrophilic lipophilic balance (HLB) varying from 10.0 to 11.25] to be highly water soluble. Introduction of these hydrophobic groups gives the polymer surface activity and unique hydration-dehydration characteristics [6]. Cellulose ethers show a limited solubility in organic solvents and their organosolubility decreases along with the hydrophobicity of METHOCEL cellulose ethers in the following order: J > E > F > A > K [4]. Upon heating, cellulose ethers dehydrate, forming a gel. Gel temperature is also strongly determined by the substitution pattern. The strength of gel increases with: (a) higher methoxy substitution; (b) lower hydroxypropyl substitution; (c) higher temperature; (d) higher concentrations and (e) higher molecular weight [4].

2.2. Hydroxypropyl cellulose (HPC) and hydroxyethyl cellulose (HEC)

Hydroxypropyl cellulose (HPC) is another cellulose-based polymer with both water solubility and organic solubility and applications in pharmaceutical field. The derivatization of cellulose is conducted using propylene oxide as alkylating agent. Although there are three hydroxyl groups per glucose unit susceptible of being etherified, the final number of hydroxypropyl moieties per glucose ring can be higher than 3 (moles of substitution, MS) if exhaustive etherification of hydroxyl groups from hydroxypropyl moieties, already incorporated, takes place. The MS value determines the solubility of the material in aqueous and organic solvents. Thus, HPC will reach a good solubility in water when MS is ≈ 4. The glass transition temperature (Tg) of HPC is approximately 120 °C. Since HPC comprises a combination of hydrophobic and hydrophilic groups, hydrophobic and hydrophilic drugs can be included in HPC matrices. In addition, HPC displays a lower critical solution temperature (LCST) at 45 °C so as, at temperatures below the LCST, HPC is readily soluble in water; above the LCST, HPC is not water soluble. It is available in different grades with varying viscosities and molecular weights ranging from 40,000 to 1,150,000 Daltons. One of the most commonly used grades of the polymer is HPC MF grade, where M indicates the viscosity type and F indicates that it is of pharmaceutical grade (Brookfield viscosity at 2% in water: 4000-6500 mPa·s; molecular weight: 850,000 Daltons) [7].

Among the cellulose ether polymers MC, HPMC, NaCMC and HPC, HPC had the lowest true density value indicating the presence of comparatively higher number of possible enclosed voids and hence, floatability if needed [8].

2.3. Carboxymethyl cellulose (CMC)

CMC is prepared by soaking crude cellulose in sodium hydroxide, and then making it react to sodium chloroacetate. Since the reaction occurs in an alkaline medium, the product is the sodium salt of the carboxylic acid ($R-O-CH_2COONa$) to form sodium carboxymethylcellulose (NaCMC); this is the reason why CMC is often used as its sodium salt. Due to the incorporation of the polar and ionizable carboxyl groups, this cellulose derivative is water soluble and sensitive to changes in pH. These materials are water soluble as long as a fraction of the carboxylic groups are ionized. This is marked by its pK_a (pK_a = 4.0 [9]). Hence, CMC aqueous solutions are stable at pH preferably over 2.5. Below pH 2, precipitation of a solid occurs due to the bulk insolubility of the practically full non-ionized CMC, in agreement with its pK_a. On the other hand, NaCMC is insoluble in organic

solvents and reacts with heavy metal salts to form films that are insoluble in water, transparent, relatively tough, and unaffected by organic materials [10].

CMC is commercialized with different molecular weights, ranging from 21,000 to 500,000, and degree of substitution. Their 1% solutions show viscosity values that vary from 5 to 2,000 mPa s, depending on the extent of functionalization, pH and molecular weights. The pH of these solutions ranges from pH 6.5 to 8 and they display a tensile strength of 8,000 to 15,000 psi [10].

2.4. Crosslinked polyacrylates: Carbomers, Carbopol®, Polycarbophyl (PCP)

Carbopol and calcium Polycarbophil present considerable differences regarding their calorimetric and mechanical behavior. Carbopol varieties showed a main glass transition around 130–135 °C, which is not dependent on their cross-linking degree and molecular weight. In contrast, in calcium Polycarbophil calcium ions act as ionic cross-linkers of the carboxylic groups, providing rigid networks with much higher Tg, and storage and loss moduli. The Tg of calcium Polycarbophil is not detected below 350 °C [11]. Gómez-Carracedo et al. also found changes particularly intense in the storage, G′, and loss, G′′, moduli at temperatures around their Tg for Carbopol samples. At temperatures below Tg, all of them showed high values of storage and loss moduli. Glass transition caused G′ and G′′ to decrease by about three orders of magnitude.

The application of carbomers can take place in a broad pH range (4.5–10). Since they are stable upon heating, their formulations can be autoclaved. Carbomer aqueous formulations display an elasto-viscoplastic behavior as well as a remarkable stability over time (no aging). The elastic effects are dominant when the microstructure is fully structured, while viscous effects dominate after yielding [12]. Their suspensions are sensitive to electrolytes (mainly multivalent cations) causing a reduction in viscosity. In contrast, the combination with propylene glycol or glycerin increases the gel viscosity. Carbomers are used as thickeners, emulsion stabilizers, gel formers, and suspending agents.

Carbopol® 971P and 71G varieties have higher molecular weight between adjacent cross-links, and conversely lower cross-linker densities (loose fishnet conformation) than Carbopol® 974P, 934 and 934P varieties (fuzzball structure). Although the determination of the total molecular weight of cross-linked polymers is quite complex compared to linear polymers, values of about 1.25×10^6 (Carbopol® 971P and 71G) and 3×10^6 (Carbopol® 974P, 934 and 934P) have been reported [11]. Molecular weights of PCP are about 7×10^5 Da [13].

2.5. Poloxamer. PEO-PPO-PEO triblock-copolymers

The European Pharmacopeia catalogues several grades of Poloxamers that are recorded in Table S9.

Table S9. Chemical composition and properties of Poloxamer grades [14].

Poloxamer type	Ethylene oxide units (a)	Propylene oxide units (b)	Content of oxyethylene (%)	Molecular weight (Da)
124	10 - 15	18 - 23	44.8 - 48.6	2090 - 2360
188	75 - 85	25 - 30	79.9 - 83.7	7680 - 9510
237	60 - 68	35 – 40	70.5 - 74.3	6840 - 8830
338	137 - 146	42 - 47	81.4 - 84.9	12700 -17400
407	95 - 105	54 - 67	71.5 - 74.9	9840 - 14 600

2.6. Alginate salts

They are arranged in blocks of M fragments, G fragments, and alternating G and M fragments. The amount and distribution of each monomer depends on the species, location, and age of seaweed from which the alginate has been isolated [15]. Several alginate salts are commercially available, being sodium alginate (NaAlg) the most commonly used. It has been reported that the chemical composition of alginates affects their compression behavior (for tablet preparation), where alginates with low G content behave more elastically than alginates with low M content. In addition, the

plasticity of potassium alginates is higher than that of sodium alginates. However, alginates deform elastically [16].

Alginates are established among the most versatile biopolymers, used in a wide range of applications and are extensively as a gelling agent in food industry, and excipient in drug products, applications generally linked to their thickening, gel-forming, and stabilizing properties [17], for example, in the formulation of GRDDS [18].

Alginic acid is a weak acidic material with a pK_a of 3.38 to 3.65 [19]. Slowly soluble in water, it forms a viscous colloidal solution at pH above 3 and remains insoluble in aqueous solutions in which the pH is lower than 3 (i.e., when its non-ionized form is predominant). This polysaccharide is virtually insoluble in ethanol (95%), diethyl ether, chloroform, and in ethanol/water mixtures in which the ethanol content is greater than 30% [20]. NaAlg forms a viscous gel layer upon contact with gastric fluids useful in low-density dosage formulations. Various grades of sodium alginate, able to yield aqueous solutions of varying viscosities, are commercially available. Typically, a 1% w/v aqueous solution, at 20°C, will have a viscosity of 20–400 mPa s (20–400cp). Viscosity may vary depending upon concentration, pH, temperature, or the presence of metal ions. Above pH 10, viscosity decreases [20].

References

1. Repka, M.A.; McGinity, J.W. Bioadhesive properties of hydroxypropylcellulose topical films produced by hot-melt extrusion. *Journal of Controlled Release* **2001**, *70*, 341–351, doi:10.1016/S0168-3659(00)00365-5.
2. Matharu, A.S.; Motto, M.G.; Patel, M.R.; Simonelli, A.P.; Dave, R.H. 2.- Evaluation of hydroxypropyl methylcellulose matrix systems as swellable gastro-retentive drug delivery systems (GRDDS). *Journal of Pharmaceutical Sciences* **2011**, *100*, 150–163, doi:10.1002/jps.22252.
3. Siepmann, J.; Peppas, N.A. Modeling of drug release from delivery systems based on hydroxypropyl methylcellulose (HPMC). *Advanced Drug Delivery Reviews* **2012**, *64*, 163–174, doi:10.1016/j.addr.2012.09.028.
4. Methocel. Available online: https://www.industrialcellulosics.com/products/methocel (Accessed on 8 May 2020).
5. Using Dow Excipients for Hydrophilic Matrix Systems. Available on-line: https://www.pdf-book-search.com/dow/using-dow-excipients-for-controlled-release-of-drugs-in.html. (Accessed on 21 June 2020).
6. Sarkar, N.; Walker, L.C. Hydration—dehydration properties of methylcellulose and hydroxypropylmethylcellulose. *Carbohydrate Polymers* **1995**, *27*, 177–185, doi:10.1016/0144-8617(95)00061-B.
7. Vo, A.Q.; Zhang, J.; Nyavanandi, D.; Bandari, S.; Repka, M.A. Hot Melt Extrusion paired Fused Deposition Modeling 3D Printing to Develop Hydroxypropyl Cellulose based Floating Tablets of Cinnarizine (in press). *Carbohydrate Polymers* **2020**, 116131, doi:10.1016/j.carbpol.2020.116131.
8. Nerurkar, J.; Jun, H.W.; Price, J.C.; Park, M.O. Controlled-release matrix tablets of ibuprofen using cellulose ethers and carrageenans: effect of formulation factors on dissolution rates. *European Journal of Pharmaceutics and Biopharmaceutics* **2005**, *61*, 56–68, doi:10.1016/J.EJPB.2005.03.003.
9. Bouchard, D.C.; Lavy, T.L. Hexazinone Adsorption-Desorption Studies with Soil and Organic Adsorbents. *Journal of Environmental Quality* **1985**, *14*, 181–186, doi:10.2134/jeq1985.00472425001400020006x.
10. Ghannam, M.T.; Esmail, M.N. Rheological properties of carboxymethyl cellulose. *Journal of Applied Polymer Science* **1997**, *64*, 289–301, doi:10.1002/(SICI)1097-4628(19970411)64:2<289::AID-APP9>3.0.CO;2-N.
11. Gómez-Carracedo, A.; Alvarez-Lorenzo, C.; Gómez-Amoza, J.L.; Concheiro, A. Glass transitions and viscoelastic properties of Carbopol® and Noveon® compacts. *International Journal of Pharmaceutics* **2004**, *274*, 233–243, doi:10.1016/j.ijpharm.2004.01.023.
12. Varges, P.R.; Costa, C.M.; Fonseca, B.S.; Naccache, M.F.; De Souza Mendes, P.R. Rheological characterization of carbopol ® dispersions in water and in water/glycerol solutions. *Fluids* **2019**, *4*,

doi:10.3390/fluids4010003.

13. Clausen, A.E.; Kast, C.E.; Bernkop-schnürch, A. The Role of Glutathione in the Permeation Enhancing Effect of Thiolated Polymers. *Pharmaceutical Research* **2002**, *19*, 602–608.
14. POLOXAMERS. In *European Pharmacopeia, 5.0*; 2005; pp. 2264–2265.
15. Lu, L.; Liu, X.; Tong, Z. Critical exponents for sol-gel transition in aqueous alginate solutions induced by cupric cations. *Carbohydrate Polymers* **2006**, *65*, 544–551, doi:10.1016/j.carbpol.2006.02.010.
16. Rahim, S.A.; Carter, P.A.; Elkordy, A.A. Design and evaluation of effervescent floating tablets based on hydroxyethyl cellulose and sodium alginate using pentoxifylline as a model drug. *Drug Design, Development and Therapy* **2015**, *9*, 1843–1857, doi:10.2147/DDDT.S78717.
17. Niranjanbhai, B.D.; Mahendrakumar, V.N.; Surendran, C.S.; Viral H, S.; UM, U. 10.- Advances in GRDDS: Raft Forming System a Review. *Journal of Drug Delivery and Therapeutics* **2012**, *2*, 123–128, doi:10.22270/jddt.v2i5.228.
18. Ramesh Babu, V.; Krishna Rao, K.S.V.; Sairam, M.; Vijaya Kumar Naidu, B.; Hosamani, K.M.; Aminabhavi, T.M. pH sensitive interpenetrating network microgels of sodium alginate-acrylic acid for the controlled release of ibuprofen. *Journal of Applied Polymer Science* **2006**, *99*, 2671–2678, doi:10.1002/app.22760.
19. Simsek-Ege, F.A.; Bond, G.M.; Stringer, J. Polyelectrolye complex formation between alginate and Chitosan as a function of pH. *Journal of Applied Polymer Science* **2003**, *88*, 346–351, doi:10.1002/app.11989.
20. Zubedi, S.S.; Mohammed, S. b) Floating Tablets and Its Polymers. *Journal of Drug Delivery and Therapeutics* **2018**, *8*, 16–24, doi:10.22270/jddt.v8i5-s.1928.
